# Coevolved Mutations Reveal Distinct Architectures for Two Core Proteins in the Bacterial Flagellar Motor

**DOI:** 10.1371/journal.pone.0142407

**Published:** 2015-11-12

**Authors:** Alessandro Pandini, Jens Kleinjung, Shafqat Rasool, Shahid Khan

**Affiliations:** 1 Department of Computer Science and Synthetic Biology Theme, Brunel University London, Uxbridge UB8 3PH, United Kingdom; 2 Mathematical Biology, Francis Crick Institute, Ridgeway, Mill Hill, London NW7 1AA, United Kingdom; 3 Department of Biochemistry, McGill University, Montreal, QC H3G 1Y6, Canada; 4 Molecular Biology Consortium, Lawrence Berkeley National Laboratory, Berkeley, CA 94720, United States of America; Monash University, AUSTRALIA

## Abstract

Switching of bacterial flagellar rotation is caused by large domain movements of the FliG protein triggered by binding of the signal protein CheY to FliM. FliG and FliM form adjacent multi-subunit arrays within the basal body C-ring. The movements alter the interaction of the FliG C-terminal (FliG_C_) “torque” helix with the stator complexes. Atomic models based on the *Salmonella entrovar* C-ring electron microscopy reconstruction have implications for switching, but lack consensus on the relative locations of the FliG armadillo (ARM) domains (amino-terminal (FliG_N_), middle (FliG_M_) and FliG_C_) as well as changes during chemotaxis. The generality of the *Salmonella* model is challenged by the variation in motor morphology and response between species. We studied coevolved residue mutations to determine the unifying elements of switch architecture. Residue interactions, measured by their coevolution, were formalized as a network, guided by structural data. Our measurements reveal a common design with dedicated switch and motor modules. The FliM middle domain (FliM_M_) has extensive connectivity most simply explained by conserved intra and inter-subunit contacts. In contrast, FliG has patchy, complex architecture. Conserved structural motifs form interacting nodes in the coevolution network that wire FliM_M_ to the FliG_C_ C-terminal, four-helix motor module (C3-6). FliG C3-6 coevolution is organized around the torque helix, differently from other ARM domains. The nodes form separated, surface-proximal patches that are targeted by deleterious mutations as in other allosteric systems. The dominant node is formed by the EHPQ motif at the FliM_M_FliG_M_ contact interface and adjacent helix residues at a central location within FliG_M_. The node interacts with nodes in the N-terminal FliG_c_ α-helix triad (ARM-C) and FliG_N_. ARM-C, separated from C3-6 by the MFVF motif, has poor intra-network connectivity consistent with its variable orientation revealed by structural data. ARM-C could be the convertor element that provides mechanistic and species diversity.

## Introduction

Bacterial motility and chemotaxis have been studied extensively for the past few decades. These studies have established two fundamental tenets: 1. the rotation of flagellar motors is energized by membrane ion potentials [1], 2. a signal phospho-relay built around a diffusible, phospho-protein CheY couples chemoreceptor state [2] to flagellar motor response. Changes in chemoreceptor state triggered by chemotactic stimuli alter motor counter-clockwise (CCW) / clockwise (CW) rotation bias, but do not affect energization of motor rotation. The binding of the CheY signal protein to FliM subunits within the rotor results in large domain movements of the adjacent FliG subunits. FliM and FliG multi-subunit organization and domain interactions are critical to understanding how the movements underlie motor response.

**Fig 1:**
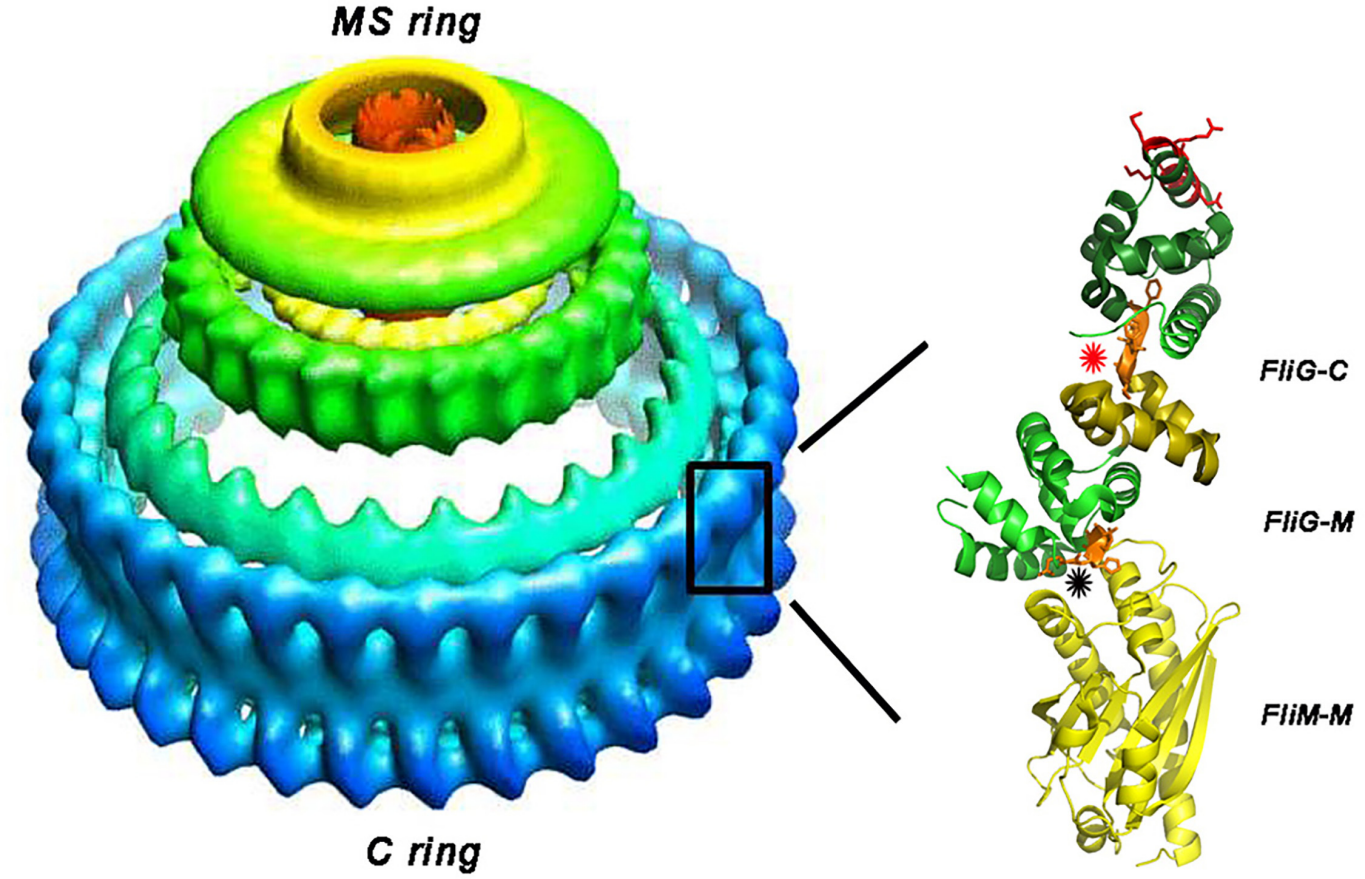
Architecture of the *Salmonella* flagellar basal body. Model of the 3D EM reconstruction (*http*:*//www*.*ebi*.*ac*.*uk/pdbe/entry/EMD-1887*) shows MS ring (green) and C-ring (blue). The MS-ring is embedded in the cytoplasmic membrane while the C-ring protrudes into the cytoplasm. There is a mismatch between the MS-ring and C-ring symmetry. Rectangle denotes likely position of the FliM_M_ FliG_MC_ complex (4FHR.pdb) in the C-ring half proximal to the MS-ring. The complex comprises FliM_M_ (yellow), FliG_M_ (green), FliG_C_ ARM-C (olive) and C1-6 terminal six-helix bundle (dark green). FliG_M_ consists of ARM-M plus a partially resolved linker. C3-6 = C1-6 four–terminal helices. Orange segments denote EHPQ (black asterisk) and MFVF (red asterisk) motifs. Charged residues on the torque helix within C3-6 are highlighted (red sidechains). The distal C-ring is comprised of the FliM C-terminal domain and FliN. S1 Fig has secondary structure nomenclature.

The C-ring, a large multi-subunit assembly within the flagellar basal body composed of the proteins FliG, FliM and FliN, forms the rotor of the bacterial flagellar motor. The C-ring architecture of isolated *Salmonella enterica* serovar Typhimurium (“*Salmonella*”) basal bodies has been determined by electron microscopy [3]. Atomic models of C-ring architecture, with implications for the switching mechanism, have been developed. The models dock the X-ray structures of the protein components into the electron microscopy reconstruction, guided by cross-link data and mutant analysis [4–6] (**Fig 1**). The switching of *Salmonella* flagellar rotation sense is “ultra-sensitive”, with a high Hill co-efficient for the activated CheY concentration *in vivo* [7] consistent with the multiple subunits [8–11]. In addition to the X-ray structures [6,12–16], NMR of isolated FliG, FliM and CheY complexes have described the protein-protein interactions affected by CheY binding [17]. CheY binds to other sites on FliM and / or FliN once tethered to FliM_N_ [17,18]. The conformational changes triggered by CheY binding could be enhanced by FliM self-association mediated by the pseudo-symmetric 3-layered α/β/α sandwich middle domain (FliM_M_) [5]. FliM_M_ and the FliG middle domain (FliG_M_) may form the gearbox that relays these changes to FliG_C_. The penultimate helix, henceforth termed “torque helix”, forms a prominent surface ridge in the FliG C-terminal domain (FliG_C_). The FliG protein has a N-terminal domain (FliG_N_) in addition to FliG_M_ and FliG_C_, all composed of multiple armadillo (ARM) repeats [6]. The torque helix interacts with the stator Mot complexes [19] and changes orientation during chemotactic stimulation [15,20]. Conserved residues identified from hidden Markov models (HMMs) of Pfam multiple sequence alignments (MSAs) (shown in *http*:*//pfam*.*xfam*.*org/clan/FliG* *)* include three short sequences (“motifs”). These motifs are GGXG in FliM_M_, EHPQ in FliG_M_, MFXF in FliG_C_ (all letters, except X, specify the conserved amino acid; while X denotes variable residue positions). FliG_C_ may be divided into an N-terminal helical triad (ARM-C) and a C-terminal six-helix bundle (C1-6) based on its flexibility around the MFXF motif in *H*. *pylori* [15]. The conservation of charged residues in the torque helix, while not absolute, has been noted [12]. The motifs are among the sites that upon mutagenesis yield CW or CCW chemotactic (*che*) phenotypes [21,22], reviewed in [6,23].

In spite of the above-noted advances, a complete atomic level knowledge of the switching mechanism has not been possible, even for the enteric *Salmonella* and *Escherichia coli* that have been the focus of studies thus far. This is due to several factors. **1**. The limited resolution of the electron microscopy reconstruction makes consensus on subunit stoichiometry or contacts difficult [24]. **2.** *Thermotoga maritima*, *Aquifex aeolicus* and *Helicobacter pylori* FliG X-ray structures used for the atomic model show the protein adopts multiple conformations [15]; while basal bodies from these and other species differ from *Salmonella* in C-ring size [25,26]. Even within one species, C-ring architecture is likely to be altered by adaptive changes [27]. **3.** Residue conservation identifies important residues but not the interactions between residue positions required for deciphering the allosteric network involved in the switching mechanism. **4**. The C-ring protein-protein interactions documented by NMR and in-situ cross-linking do not fully agree [28]. **5**. The chemotactic response of the flagellar motor differs between species. While CheY binding switches rotation sense from CCW to CW in the enteric bacteria; this logic is inverted in *Bacillus subtilis* [29]. In *Rhodobacter sphaeroides* and *Sinorhizobium mellioti*, the motor alternates between rotation stops and starts [30–32]. CheY is dephosphorylated at the motor by FliY [33], present with, or instead of, FliN in many species [34]. Part of FliY is homologous to FliM_M_. This FliY segment could complement or substitute for FliM_M_ interactions with FliG in gram positives. Thus, even if complete knowledge of the switching mechanism were achieved for *Salmonella*, its general applicability would remain an issue.

We present, here, a novel approach based on covariance analysis of coevolved mutations [35] for identification of the common design principles of the flagellar motor switch. The method has important advantages. First, in common with residue conservation, its conclusions are based on a wide database and, therefore, have generality. Second, it records interactions at single residue detail. This is true also for NMR, but only for isolated complexes of limited size, and in-situ crosslinking, but only for positions selected for study. The disadvantages are analysis and interpretation of the large amount of information contained in a coevolution matrix. We developed metrics based on network tools [36] to make the analysis tractable and mapped the correlations onto the atomic structures to facilitate interpretation. We find that FliM_M_ has an unusually compact coevolution network, a feature that is explained by the primacy of the inter-subunit contacts for FliM self-association. FliM_M_ and the FliG_C_ terminal four-helix bundle (C3-6), built around the torque helix, communicate via an allosteric network mediated by a few surface-proximal patches in FliG organized around the EHPQ motif. The patches are targeted by deleterious *che* mutations, underlining the importance of the network for signal transduction in the switch complex.

## Results

**Fig 2:**
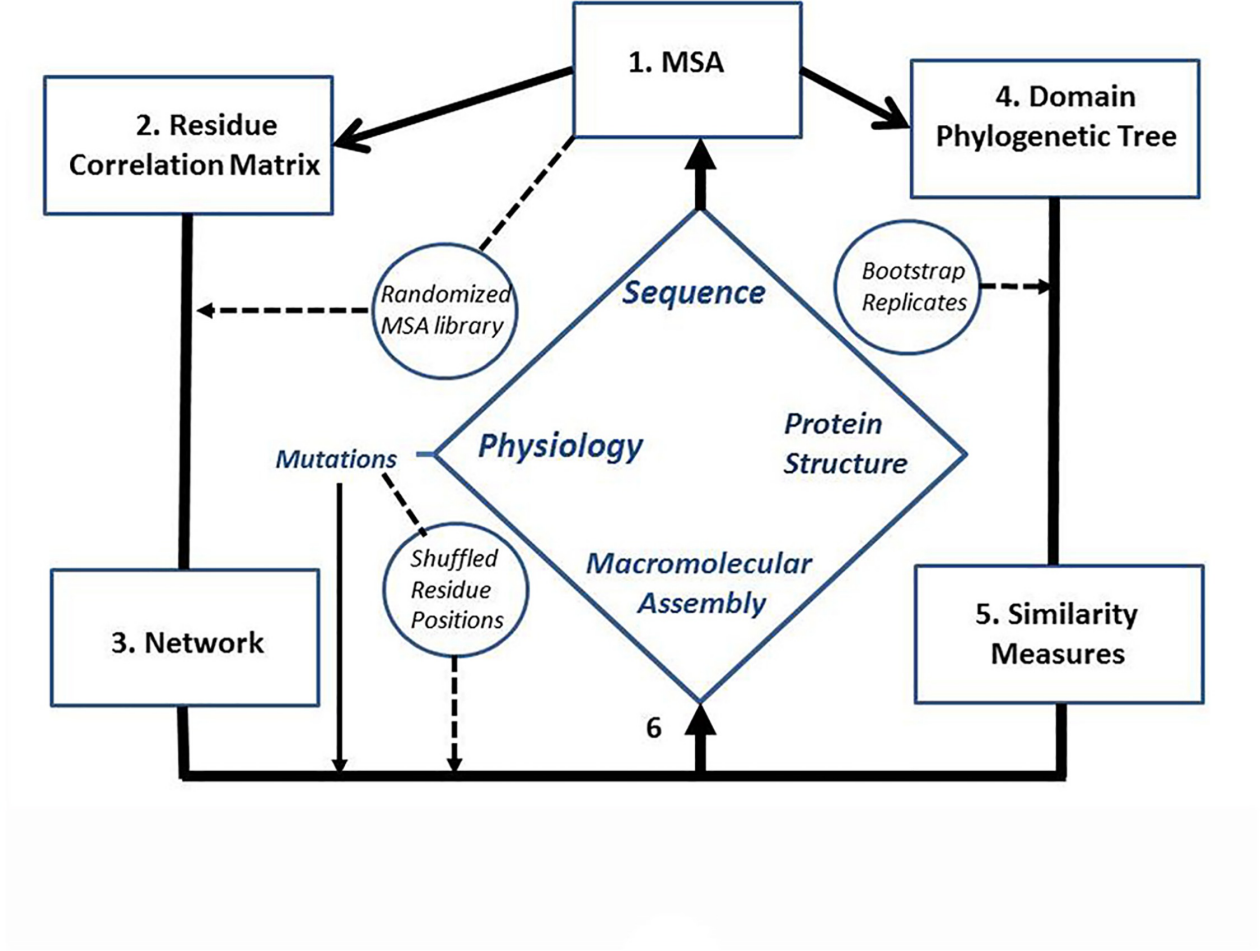
Computational Strategy. The experimental data obtained on the system are enclosed within the central blue diamond. **1.** Multiple sequence alignments (MSAs) were formed from the amino acid sequences. The MSAs were the basis for all computations. **2**. They were used for construction of residue coevolution matrices. **3**. The matrices were represented and analysed as a network. **4**. The MSAs were also used to construct phylogenetic trees of individual domains. **5**. The trees were compared with similarity measures to detect domain coevolution. **6.** The results were integrated with the X-ray protein structure and *in-situ* cross-linking data to infer FliG and FliM subunit interactions in the intact basal body. Randomized MSA libraries, shuffled mutation lists and bootstrap replicates assessed statistical significance.

**Fig 2** gives an overview of the computational strategy. **1.** MSAs of FliG and FliM were the basis for all analysis. The information content and conservation score for residue positions was determined to guide subsequent steps. The MSAs were mapped onto structures for identification of conserved surface residues potentially involved in inter-domain interactions. **2.** Correlations between residue positions were the main measure of coevolution. We created randomized MSA libraries to estimate the statistical significance of the correlations. The original coevolution matrices were compared against the population of correlation matrices generated from the randomized libraries. Lists of chemotactic mutations in *Salmonella* based on swarm plate assays were matched to the residue correlation network. The lists were shuffled to score for random matches. **3.** A network model of the original coevolution matrices was generated and metrics developed to measure residue, patch and domain coevolution. **4**. Phylogenetic tree similarity provided an alternate check for domain coevolution. Replicates were used to assess the robustness of the most likely phylogenetic tree for each domain. **5.** Phylogenetic tree topologies were compared by computation of the fit probabilities of the domain MSAs with a reference domain phylogenetic tree. **6.** The results were evaluated in the context of available structural knowledge. Custom scripts to perform various tasks were written in C, python and R (*http*:*//www*.*r-project*.*org*). They are available upon request. Procedures for each step are detailed in Methods.

### FliM_M_ contacts dominate the FliM_M_FliG_MC_ coevolution matrix

A coevolution matrix contains a large number of correlations between residue positions. The numbers scale as the square of the protein sequence (e.g. 10^4^ possible correlations for a 100 residue protein). The correlations fall into three categories; residual correlations due to finite MSA depth and diversity, correlations due to residue contact either within or between domains and long-range correlations due to allosteric couplings.

Our analysis is based on representation of the coevolution matrix as a network, with residue positions as “nodes” and the correlations between them as “edges”. The contribution of residue positions to the network is then obtained as their centrality [37]. The eigenvector centrality, *E*, is calculated directly from the correlation matrix:

$$E.{\left(M\;\right)}_{evol}=E.\lambda$$ Eq 1

where (*M*)_*evol*_ is the coevolution matrix and λ the corresponding eigenvalue. We define the mean centrality of “*i*” residue positions as their weight. $W=\sum_{i=1}^{n}\;E/n$. The number of contiguous residue positions, *n*, is 6, unless otherwise noted. “Node” will henceforth refer to such six-residue segments in the complete network or its derived sub-networks, rather than individual residues. The weight W measures the network information content contained in a node. It is a product of the mean strength of the correlations formed by the node with other nodes times the number of correlations or its connectivity. Domain-level measures for correlation strength (*S*_*M*_) and connectivity (*C*) are defined later in this Section.

**Fig 3:**
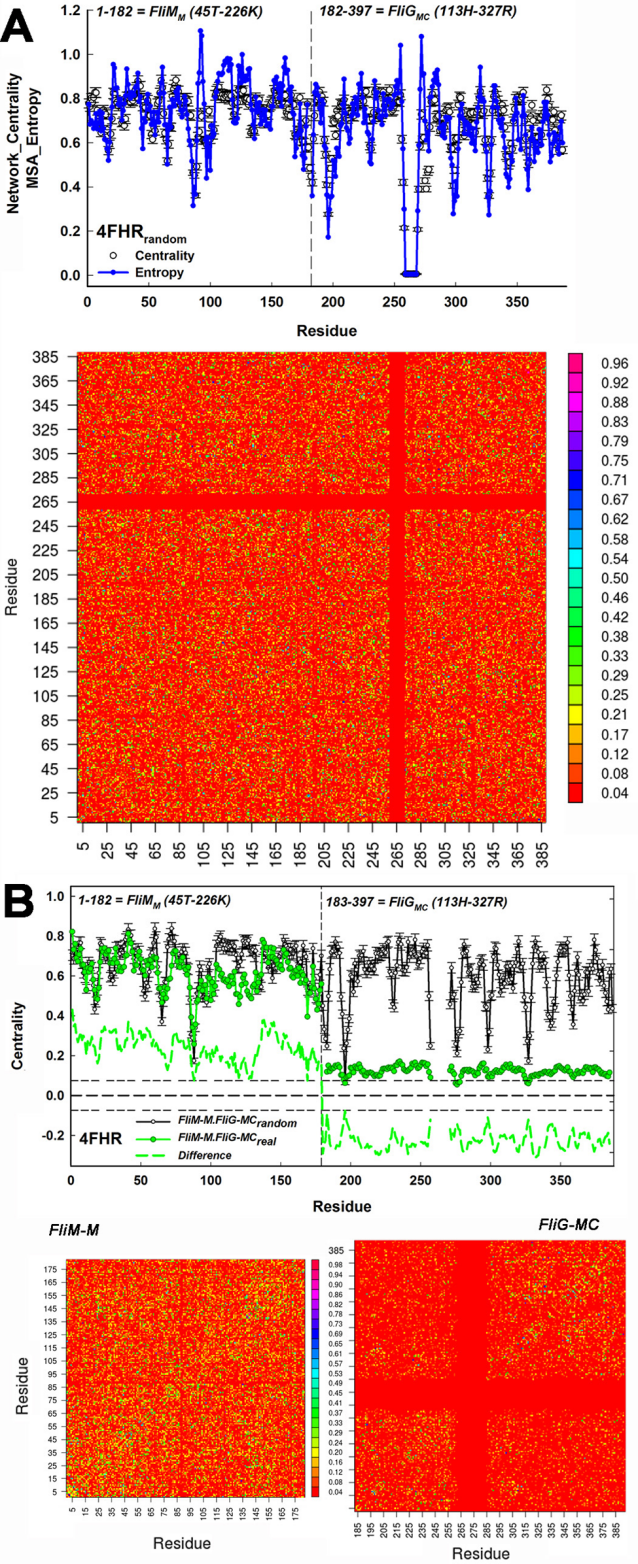
FliM_M_ dominates FliG_MC_ in the composite FliM_M_.FliG_MC_ network: Dashed vertical lines in plots denote the boundary between FliM_M_ and FliG_MC_. Residue positions in the concatenated *T*. *maritima* FliM_M_FliG_MC_ 4FHR.pdb MSA are on the X-axes. (**A) Top**. The mean randomized library centrality profile (±σ) of the network representation (open circles) of the matrices from the shuffled FliM_M_.FliG_MC_ MSA library. The MSA entropy (blue line), unaltered by the shuffling procedure, is superimposed to show that the entropy determines the residual correlations reflected in the centrality. Residue positions 259–270 (*T*. *maritima* FliG 187F-198I)) have low entropy as they are absent from many species. **Bottom.** One shuffled matrix. The matrix is mirror-symmetric about the positive diagonal with positive correlations distributed over the matrix, except segment 259–270. Vertical colour bar denotes normalized correlation value (0–1). (**B) Top**. The composite network centrality profile, together with the difference profile obtained after correction for the residual correlations. Horizontal short dashed lines represent (±σ) variation around the zero mean of the difference profile expected from residual correlations (thick dashed line) **Bottom.** The FliM_M_ and FliG_MC_ coevolution matrices show that FliM_M_ correlations are uniformly distributed relative to the FliG_MC_ correlations. In particular, the FliG_M_FliG_C_ inter-domain correlations (FliG_MC_ matrix top left, bottom right) are sparse relative to the intra-domain correlations. Vertical bar is as in A.

We first corrected for residual correlations in order to study the correlations due to protein domain interactions. The residual correlations were characterized by generation of a library of randomized MSAs (n = 100) in which the amino acid residues were shuffled column by column. This method preserved the entropy at residue positions. The randomized MSA library was batch-processed with the PSICOV algorithm [38] to generate a stack of randomized correlation matrices. One example of a randomized matrix is shown together with the mean centrality profile of the randomized MSA library for the FliM_M_FliG_MC_ complex (4FHR.pdb) (**Fig 3A**). The centrality of residue positions superimposed with their entropy in the MSA. The consistency between the two measures shows that the potential fractional contribution of residue positions to the network information content is given by their Shannon Entropy (Methods). The entropy differences between residue positions are created by the finite MSA size and diversity, with an extreme example of low entropy being positions occupied by only acidic (E, D) or basic (K, R) residues. In contrast, all nodes have the same entropy in an ideal random network and, thus, equal W (default value 1). Primary nodes of a coevolved network were then defined as those with W > W_MEAN+2σ_, where σ is the deviation expected from the randomized MSA library.

We now examined the real coevolution matrix of the FliM_M_FliG_MC_ complex (**Fig 3B**). We obtained the striking result, seen in the centrality profile, that FliM_M_ collectively had greater weight in the composite matrix than FliG_MC_. Inspection of the FliM_M_ and FliG_MC_ matrices revealed the reason. The FliM_M_ matrix was more densely and uniformly populated than that for FliG_MC_. The weight *δW*_*i*_ of residue position “*i"* in the difference profile was computed from the equation

δWi=Ei−(MEMR)ER Eq 2

where *E*_*i*_ and *E*_*R*_ are the real centrality and randomized library centrality at position *i*, while (MEMR) is the ratio of the real over randomized library centrality means, averaged over the profile. The difference (*δW*_*i*_) profile confirmed that the difference between the mean FliM_M_ and FliG_MC_ weights exceeded the expected deviations in the centrality profile due to network noise from residual correlations. We sought an explanation for this difference.

### Inter-subunit contact correlations account for the high-density of the FliM_M_ coevolution matrix

The high-density of the FliM_M_ matrix results from correlations between distant sequence positions. Distant sequence positions imply physical separation. If so, the density of the FliM_M_ matrix could indicate inter-subunit contacts and / or allosteric couplings. We used available structural knowledge based on cross-link data (**Table 1**) as well as the X-ray structures to evaluate these possibilities.

**Table 1:** In-situ crosslinks: REF = Reference.

FliG / FliG	REf	FliM / FliM	REF	FliM / FliG	REF
44^**”**^/148^**+2**^	[28]	56^*1*^/**93** ^*2*^ **,184** ^*5*^ **,186** ^*5*^,*192* ^*5*^	[20]	129/*219*	[20]
118^**−2**^/167,171^**3**^	[4]	57^*1*^/185^*5*^	[5]	130/203,207,**215,219**	[20]
121^**−2**^/167,171^**3**^	[4]	63^*1*^/*184* ^***5***^,*186* ^***5***^,*192* ^***5***^	[20]	140^**1**^/*207*	[20]
295^**6**^/295^**6**^	[4]	64^*1*^/94^*2*^,185^*5*^	[5]	145^**1**^/227^−4^	[20]
296^**6**^/296^**6**^	[4]	76/*184* ^***5***^	[20]	149^**+1**^/**207**	[20]
298^**6**^/298^**6**^	[4]				
281^**−6**^/298^**6**^,300^**6**^	[4]				
199,207,212,233^**4**^/315^**7**^	[15]				

**Fig 4:**
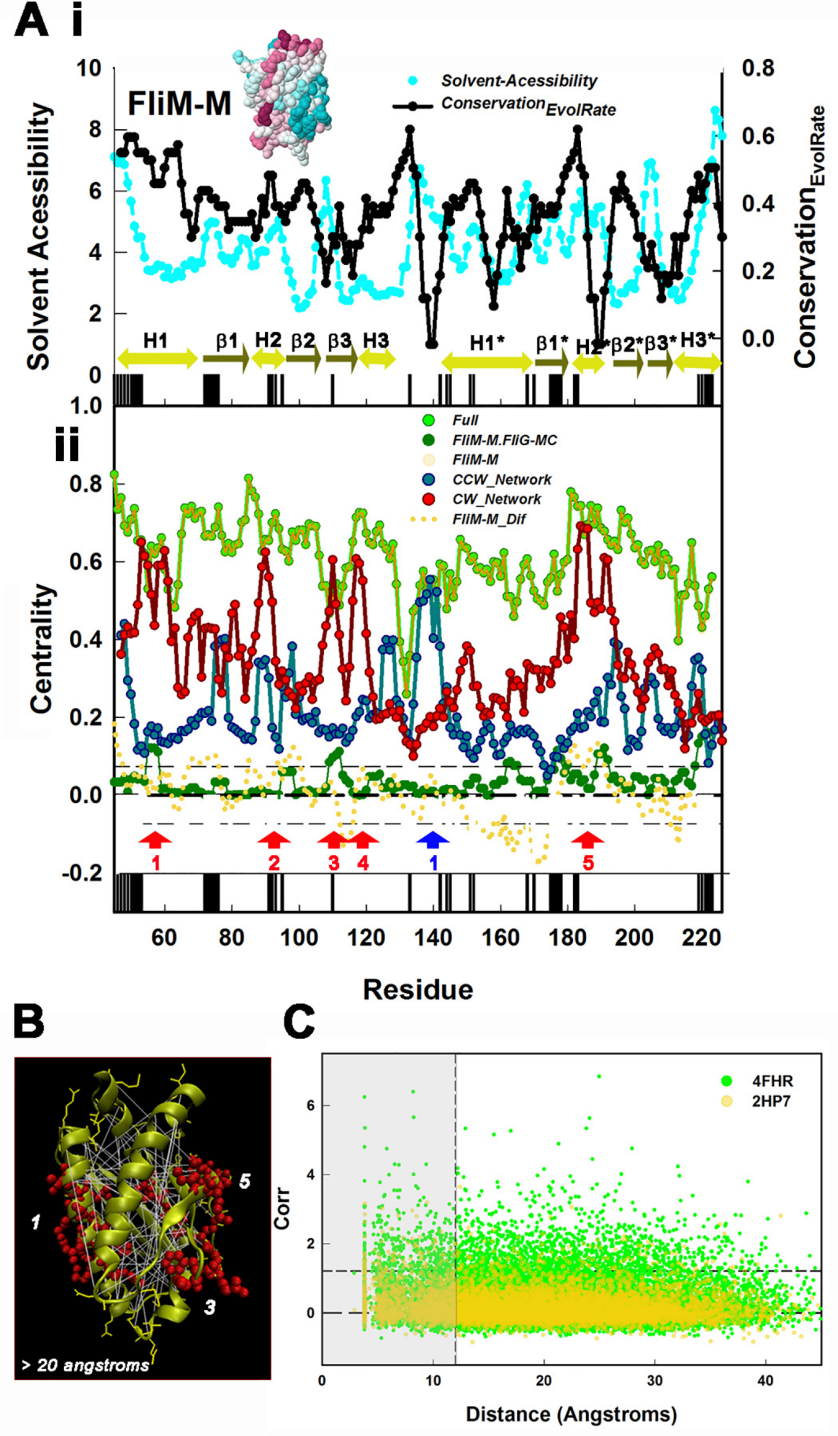
The high connectivity of the FliM_M_ network is explained by inter-subunit contacts. (**A)** Features in the FliM_M_ centrality profile were analysed by determination of conserved surface residues and maps of chemotactic mutations. **i.** Conserved surface residue positions were identified by a two-step filter based on solvent accessibility (cyan symbols) (determined from the *T*. *maritima* FliM_M_ structure (2HP7.pdb)) and conservation based on evolutionary rate (black symbols). The vertical black bars positioned along the sequence represent contiguous (>4) residue patches where both conservation and solvent accessibility exceed their respective mean values. Secondary structure elements are above the bars. H = α-helix (lime green), β = β-sheet (dark green with arrowhead). Asterisks indicate pseudo-symmetric equivalents. **Inset:** The 2HP7.pdb structure colour coded for conservation (strong (purple)–weak (blue)). **ii.** Network centrality profile of FliM_M_ alone (gold symbols) is identical to the FliM_M_ profile in the composite FliG_M_.FliG_MC_ network. Thus intra-domain correlations determine the centrality of FliM_M_ residue positions in the composite network. The horizontal short dashed lines around the zero mean difference (bold dashed line) show the (±σ) deviation expected due to residual correlations (as in 3B). There are no significant peaks in the FliM_M_ difference profile (dotted gold line) or the FliM_M_FliG_MC_ inter-domain correlations, consistent with the dominance of FliM_M_ intra-domain correlations. Centrality profiles of the CW (red) and CCW (blue) chemotactic networks show distinct CW (red arrows) and CCW (blue arrow) primary nodes. With the exception of CW node 4, the nodes are within or adjacent (< 7 residues) to the conserved surface patches. (**B)** Map of high-scoring correlations (white lines) between residue positions (gold stick side chains) in 2HP7.pdb (gold cartoon C^α^ backbone). Red spheres mark residue positions equivalent to positions targeted by CW mutations in Salmonella. Numbers mark CW primary node segments identified from the centrality profile in A. (**C)** The distribution of correlation values as a function of the C^α^-C^α^ distance between the paired residues. Shaded grey area demarcates the contact zone (< 12 angstroms). The short dashed line marks the 3σ threshold for high-scoring correlations.

Other headers denote the protein pair (“Protein1/Protein2”) whose residues are crosslinked. Crosslinked pairs are denoted as “Residue1/Residue1”, or as “Residue1/Residue1,Residue2,—”where Residue1 from Protein1 forms multiple crosslinks with Residue1,Residue2,—from Protein2. Superscript denote network nodes identified in Figs 4A and 6A that either include or are adjacent (+ = C-terminal,— = N-terminal) in the sequence to the crosslinked residue. Residue font denotes it forms a crosslink whose yield is increased by either repellent (italic) or attractant (bold) stimuli. Superscript color denotes either a FliM_M_ CW (italic) or CCW (bold) node. Residue numbers from *E*. *coli* and *H*. *pylori* have been converted to *T*.*maritima* residue numbers based on the MSA

We screened the *T*. *maritima* FliM_M_ (2HP7.pdb) for conserved surface residue positions (**Fig 4A**). We reasoned that surface residues that mediate inter-subunit contacts should be conserved for residue type (hydrophobicity or charge) relative to those that do not. The *che* mutations in *Salmonella* [21] have been proposed to target sites for FliM self-association [5]. We therefore constructed networks comprising all possible interactions between residue positions equivalent to those targeted in *Salmonella* and examined their centrality. We assumed that the correlations between the mutated positions had equivalent strength. Binary mask matrices, with same dimensionality as (*M*)_*evol*_, representing the interactions between CW or CCW mutant positions were created; with elements representing correlations between mutated positions having value 1, and other elements value 0. The correlation matrices were obtained by multiplication of [*M*]_*evol*_ by the mask matrices. The CCW mutation network had one primary node, while the CW network had several. These nodes, with two exceptions (nodes 1 and 4), mapped within or close to conserved surface residue patches (Fig 4A).

We recorded the C^α^-C^α^ physical distance separating correlated residue positions in the *T*. *maritima* FliM_M_ structures (**Fig 4B**). High-scoring correlations were mapped on the structures. A more stringent +3σ threshold (Methods) was used for the single correlations, relative to the +2σ threshold employed for the 6 residue nodes in the centrality profiles, with σ in both cases determined from the randomized libraries. Many correlations were between pairs greater than 20 angstroms apart in the FliM_M_ subunit. The residues localized at subunit surfaces marked by the CW mutations, linking positions in CW nodes 1 (H1), 3 (β2, β3) and 5 (between β1* and H2*). In-situ cross-link data have shown that these surface elements participate in inter-subunit contacts. The long-range (> 20 angstrom) correlations had comparable values to the contact (< 12 angstrom) correlations. The consequence was that correlation strength had a weak dependence on distance (**Fig 4C**). The mean value / fraction above threshold for the contact (< 12 angstrom) population is 1.74 ± 0.72 / 0.15, versus 1.66 ± 0.54 / 0.1 for the non-contact (>12 angstrom) population. The dependence was insensitive to whether FliM_M_ was in isolation, or in complex with FliG_MC_; though values were inflated for FliM_M_ correlations in the complex due to inclusion of the low-scoring FliG_MC_ correlations in the normalization. This result implies that the inter-subunit contacts are as important as the intra-subunit contacts that maintain the domain fold.

### Coevolution analysis indicates that FliM_M_ interfacial contacts for self-association are more conserved than the FliM_M_ contact with FliG_M_

**Fig 5:**
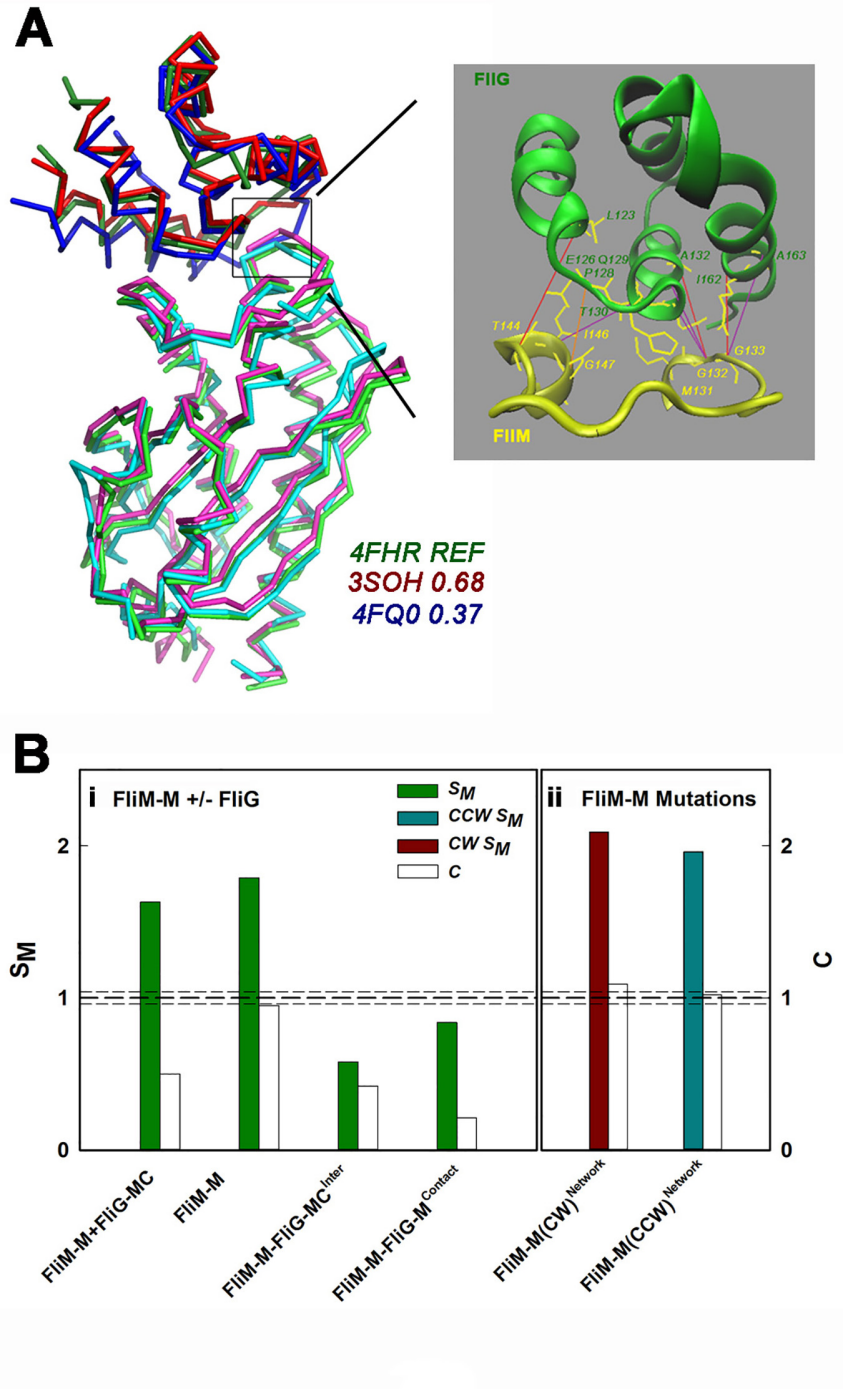
The FliM_M_FliG_M_ contact correlations are weaker than FliM_M_ inter-subunit contacts. (**A)** Superposition of the FliM_M_ and FliG_M_ C^a^ backbones of the available *T*. *maritima* (4FHR.pdb, 3SOH.pdb) and *H*. *pylori* (4FQ0.pdb) structures show a conserved FliM_M_.FliG_M_ contact (black square). RMSD (angstrom^2^) values are listed. **Inset:** The high-scoring correlations (coloured lines) between residues (numbered with yellow side-chains) mapped onto the enlarged 4FHR.pdb contact. Line colour denotes correlation strength (strong (orange / red)–weak (purple)). The correlated FliM residues cluster at two locations along the FliM_M_ loop M131-E147 (CCW node 1), namely the G_132_GXG_135_ motif and I_144_-G_147_. The correlated FliG residues in the FliG_M_ segment P116-E170 cluster at E_126_HPQ_129_ motif plus residues T310, A132 in the adjacent helix and two residues (I162, A163) in the helix neighbouring it. (**B) i.** The mean strength, S_M_ and connectivity, C of the composite FliM_M_.FliG_MC_ network, the isolated FliM_M_ network and the FliM_M_FliG_MC_ interaction network compared with that for the FliM_M_ contact with FliG_M_. **ii.** S_M_ and C of the networks constructed from residue positions equivalent to those targeted by chemotactic mutations in *Salmonella*. The values have been normalized relative to the randomized library (mean (thick dashed line) ± σ (thin dashed lines)) (see Table 2).

An alternative explanation to inter-subunit contacts is that the high-density of the FliM_M_ coevolution network results from multiple contacts between residue positions due to conformational variability between species that smear out correlations over the coevolution matrix. Superposition of the structures from the evolutionary distant *T*. *maritima* and *H*. *pylori* species does not support this explanation. The structures have a common fold (**Fig 5A**), even though there are some differences [16]. The correlation values are also too high for the multiple-fold alternative to be credible. The superposition indicates a common FliM_M_FliG_M_ contact, as well as FliM_M_ fold. In contrast to FliM_M_ self-association where residue correlations span the complete inter-subunit contact interface, the coevolution of the FliG_M_FliM_M_ contact is clustered around the conserved FliM_M_ GXGG and FliG_M_ EHPQ motifs (see Introduction) as shown by the map of the high-scoring correlations (**Fig 5A Inset**).

Conformational changes triggered by CheY need to propagate along the C-ring, as well as from its distal to proximal end. A quantitative comparison of the correlation strength of the FliM_M_ inter-subunit contacts versus the FliM_M_FliG_M_ contact could evaluate the dominance of these pathways for chemotactic signal transmission. As noted, the collective FliM_M_ W is determined by intra-domain, rather than FliG_MC_ interactions in the composite network (Fig 4A). We now developed two metrics for the interactions (“edges”) that contribute to the node weight, W.

The first metric, S_M_ is a measure of mean correlation strength.

SM=(∑Mcorr>0N+corr)/(∑Mcorr>0random/Ncorrrandom) (3)

The second metric, *C*, is a measure of connectivity

C=(NcorrNmatrix)/(NcorrrandomNmatrix) (4)

The relative strength, S_M_, and connectivity, *C*, of the networks that involve the FliM_M_ domain is shown (**Fig 5B**). The parameters used to compute these metrics from Eqs 3 and 4 are listed in **Table 2**. The calculations confirm the greater strength, S_M_, and connectivity, C, of FliM_M_ within the composite network. The C between FliM_M_ residue positions is within 5% of that obtained for the randomized networks and exceeds the C of the composite network two-fold. The S_M_ and C of the FliM_M_ correlations with FliG_MC_ are three and two-fold lower respectively than for the FliM_M_ network. Interestingly, while correlations within the FliM_M_FliG_MC_ contact have increased S_M_ relative to the overall correlations between FliM_M_ and FliG_MC_ as might be expected for contact pairs, C is two-fold lower. The latter result shows that the contact is localized, consistent with the contact map (Fig 5A inset).

**Table 2:** Parameters used for computation of the strength, S_M_, and connectivity, C, of the FliM_M_ networks.

	*ΣMatrix*_*corr>0*_	*N*_*Corr*_	*N*_*corr>0*_	*N*_*Matrix*_	*N*_*corr*_ */N*_*Matrix*_	*ΣMatrix*_*corr>0*_ */N*_*corr>0*_	*C*	*S*_*M*_
**FliM**_**M**_ **FliG**_**MCRandomized**_	17621.4	116900	53348	152100	0.77	0.33	1	1
**FliM**_**M**_ **.FliG**_**MC**_	19808.8	58534	36892	152100	0.38	0.54	0.5	1.63
**FliM**_**M**_	9776.6	24142	16538	33124	0.73	0.59	0.95	1.79
**FliG**_**MC**_	9315.9	22558	16622	43264	0.52	0.56	0.68	1.7
**FliM**_**M**_ **.FliG**_**MC**_^**inter**^	716	11834	3732	36764	0.32	0.19	0.42	0.58
**FliM**_**M**_ **FliG**_**M**_^**contact**^	11.1	136	40	825	0.16	0.28	0.21	0.84
**FliM**_**M**_ **FliG**_**C**_^**contact**^	4.8	68	30	625	0.11	0.16	0.14	0.48
**FliM**_**M**_ **(CW)** ^**Network**^	658.5	1206	860	1444	0.84	0.76	1.09	2.09
**FliM**_**M**_ **(CCW)** ^**Network**^	145.1	284	202	361	0.79	0.72	1.02	1.96

ΣMatrix_corr>0_ = Sum of all positive value correlations post-PSICOV normalization N_Corr_ = Number of matrix elements with correlation values. N_corr>0_ = Number of matrix elements with positive correlation values. N_Matrix_ = Number of matrix elements (dim1*dim2), where dim1 and dim2 are the matrix dimensions. N_corr_/N_Matrix_ = Connectivity. ΣMatrix_corr>0_/N_corr>0_ = Strength. C and S_M_ are obtained after normalization by the randomized library values as defined in Eqs 3 and 4.

The CW and CCW chemotactic networks have greater (10–20%) S_M_, than the complete FliM_M_ network from which they are derived (Fig 5B). C is also improved. The change is small since C for the complete FliM_M_ network is already ¾ of the maximum possible. Binary mask matrices (n = 1000) with elements “$\sum_{i=1}^{n}\sum_{i=1}^{n}\left(i,i+1\right)$”; of value 1, generated by permutation from the list “*I*, *i+1*, *i+2 … n*” of mutated residue positions, were used to create a population of dummy CW or CCW networks to estimate significance. The S_M_ and C of the CW dummy networks generated from the lists were 0.90±0.08 and 0.91±0.05 respectively of the real CW FliM_M_ sub-network. Thus, the CW mutations target the more prominent features of the FliM_M_ network. This is not the case for the CCW mutations. The S_M_ and C of the CCW dummy list was 1.04±0.17 and 1.00±0.10 respectively of the real CCW FliM_M_ network.

### The EHPQ motif forms the dominant primary node in the complete FliG network

We have presented thus far, evidence for extensive coevolution of FliM_M_ and localized coevolution of the FliM_M_ contact with FliG_M_. The FliG_M_ EHPQ motif was a primary node in a two-point FliM_M_FliG_M_ contact (Fig 5A). We now examined the FliG network to understand the linkage between the EHPQ motif and FliG_C_.

**Fig 6:**
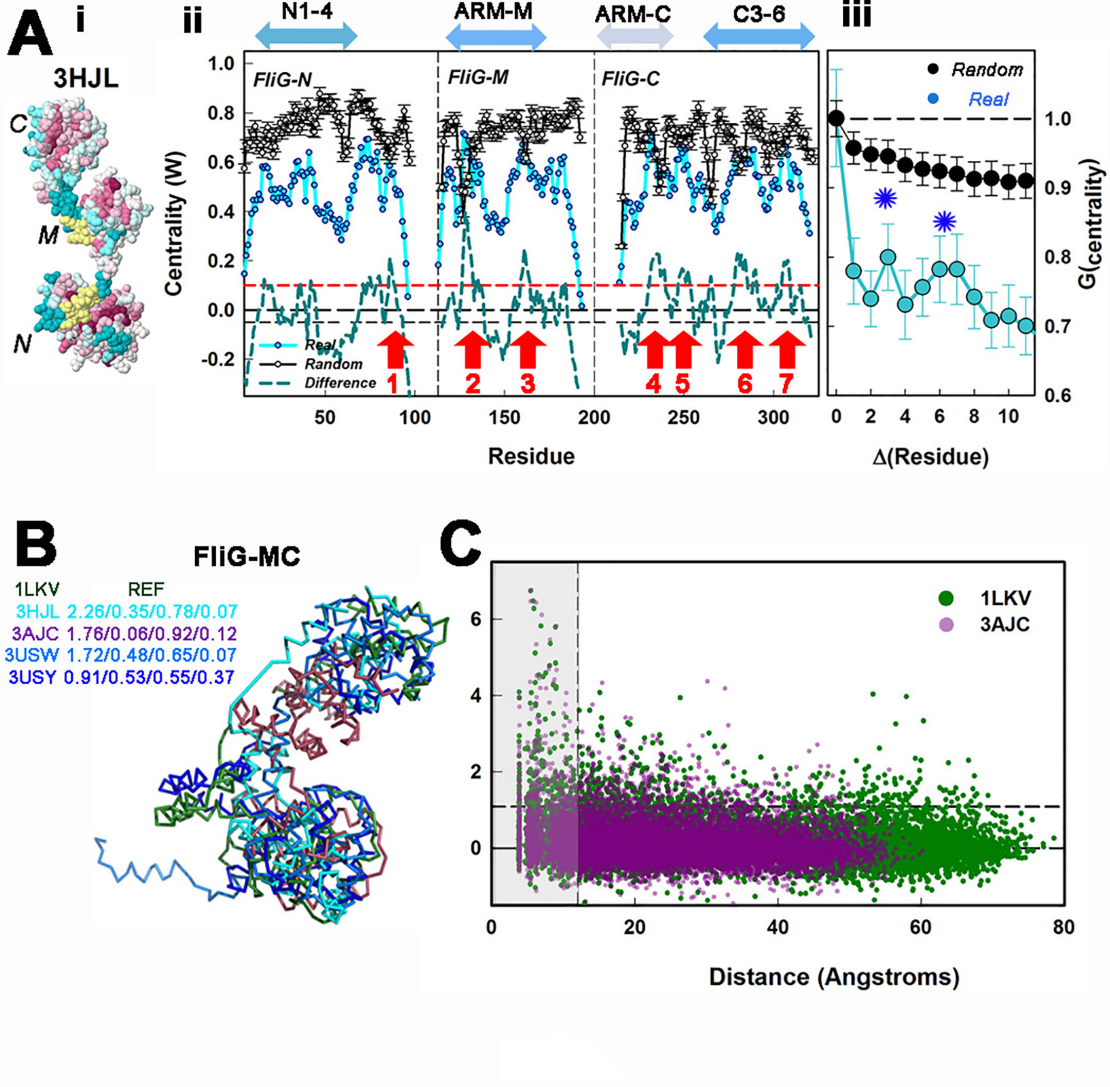
FliG network architecture. **(A) i.** *A*. *aeolicus* full-length FliG (3HJL.pdb) colour coded to show residue conservation as in Fig 4A. Segments that could not be scored are in yellow. N, M and C denote the amino-terminal, middle and carboxy-terminal domains. **ii.** FliG network centrality profile based on the trimmed 3HJL.pdb MSA, with *A*. *aeolicus* FliG residue numbers. The centrality (cyan symbols) was computed from the correlation matrix and corrected for residual correlations as for the FliM_M_FliG_MC_ complex (Fig 3B). The mean randomized MSA library (black symbols) and the corrected difference (dashed cyan line) profiles are also shown. Vertical lines delineate domains. Horizontal dashed lines mark the expected deviation due to residual correlations (+2σ (red), -σ (black)). Arrows (red) denote primary nodes. The peak modes, numbered from N to C terminal (3HJL.pdb residue positions), are FliG_N_ 86K, FliG_M_ H128 (EHPQ motif) and K161, FliG_C_ K235 (adjacent to MFXF motif), D249, S282 and Q308. The gaps in the profile are due to deletion tolerant sequence segments (yellow patches in 3HJL.pdb (colour coded for conservation as 2HP7.pdb (Fig 4Ai))). Double-arrowhead bars show sequence positions of subdomains; N1-4 = K5-K114; C3-6 = D258-D320; ARM-M = D116-L166; ARM-C = E197-F237 (3HJL.pdb residue positions). **iii.** Correlation functions (G_centrality_) as a function of residue spacing (Δ(Residue)), for the real and randomized centrality profiles. Asterisks mark peaks. (**B)** Superposition of the 5 FliG_MC_ structures. RMSD (Angstrom^2^) values are listed for the superposition of the full FliG_MC_ / ARM-M / ARM-C and C3-6. (**C)** The distance dependence of correlation values for the *T*. *maritiima* FliG_MC_ stacked (3AJC.pdb) and extended (1LKV.pdb) conformations. The stacked conformation has a smaller distance range.

The FliG coevolution matrix was generated from the MSA derived from concatenation of the Pfam FliG domain MSAs and trimmed to the full-length *A*. *aeolicus* FliG (3HJL.pdb) sequence. The three 3HJL.pdb domains and intervening linkers form 20 α-helix segments. We focus attention on four sub-domains, N1-4 (H 1–4), ARM-M (H 7–10) ARM-C (H 12–14) and C3-6 (H 17–20) whose sequence locations are shown together with the FliG centrality in **Fig 6A**. The mean W values are 0.5±0.13 (N1-4), 0.4±0.1 (C3-6), 0.33±0.14 (ARM-M) and 0.18±0.1 (ARM-C). The α-helical structure of the protein is encoded in the coevolution matrix, as revealed by peaks due to the axial 3.5 residue repeat in the auto-correlation of the centrality. The peaks are absent in the auto-correlation of the randomized MSA library. The difference centrality, corrected for residual correlations, was obtained from Eq 2. Two important conclusions result. First, FliG_N_ collectively has comparable weight, W, to FliG_MC_. Second, primary nodes can be identified in the difference profile. The EHPQ motif forms the dominant node 2 with the highest W, out of the seven nodes identified.

As for FliM_M_FliG_MC_, we used the available structures to determine the dependence of correlation strength on the physical distance between correlated residues. However, conformational heterogeneity was evident in the FliG_MC_ structures (**Fig 6B**). Superposition shows that the heterogeneity as assessed by the root mean square deviation (RMSD) is due to the inter-domain linkers, since the individual domain RMSDs are lower than the overall RMSD. Within the sub-domains, ARM-C is the most, and the C3-6 the least, heterogeneous. Short-range correlations that represent contact interactions (< 12 angstrom distance (shaded block)) were notably stronger than long-range non-contact (< 12 angstrom) correlations, as shown for the two extreme conformations (1LKV.pdb = extended, 3AJC.pdb = compact) (**Fig 6C**). Contact correlations have 30% greater strength and over two-fold greater fraction of high-scoring correlations, F (= high-scoring / total), than non-contact values. The mean strength / F for the 3AJC.pdb contact population were 2.11 ± 1.15 / 0.13, versus 1.6 ± 0.52 / 0.05 for the non-contact population. The mean strength / F for the 1LKV.pdb contact population were 2.09 ± 1.13 / 0.13, versus 1.48 ± 0.52 / 0.06 for the non-contact population.

In conclusion, the FliG network is different from the FliM_M_ network in that it has distinct maxima in the centrality profile and contact distance dependence. The FliG domains have comparable W, in the network, in contrast to FliM_M_ and FlIG_MC_ in the composite network.

### The torque helix alters the pin-wheel FliG ARM domain network architecture

**Fig 7:**
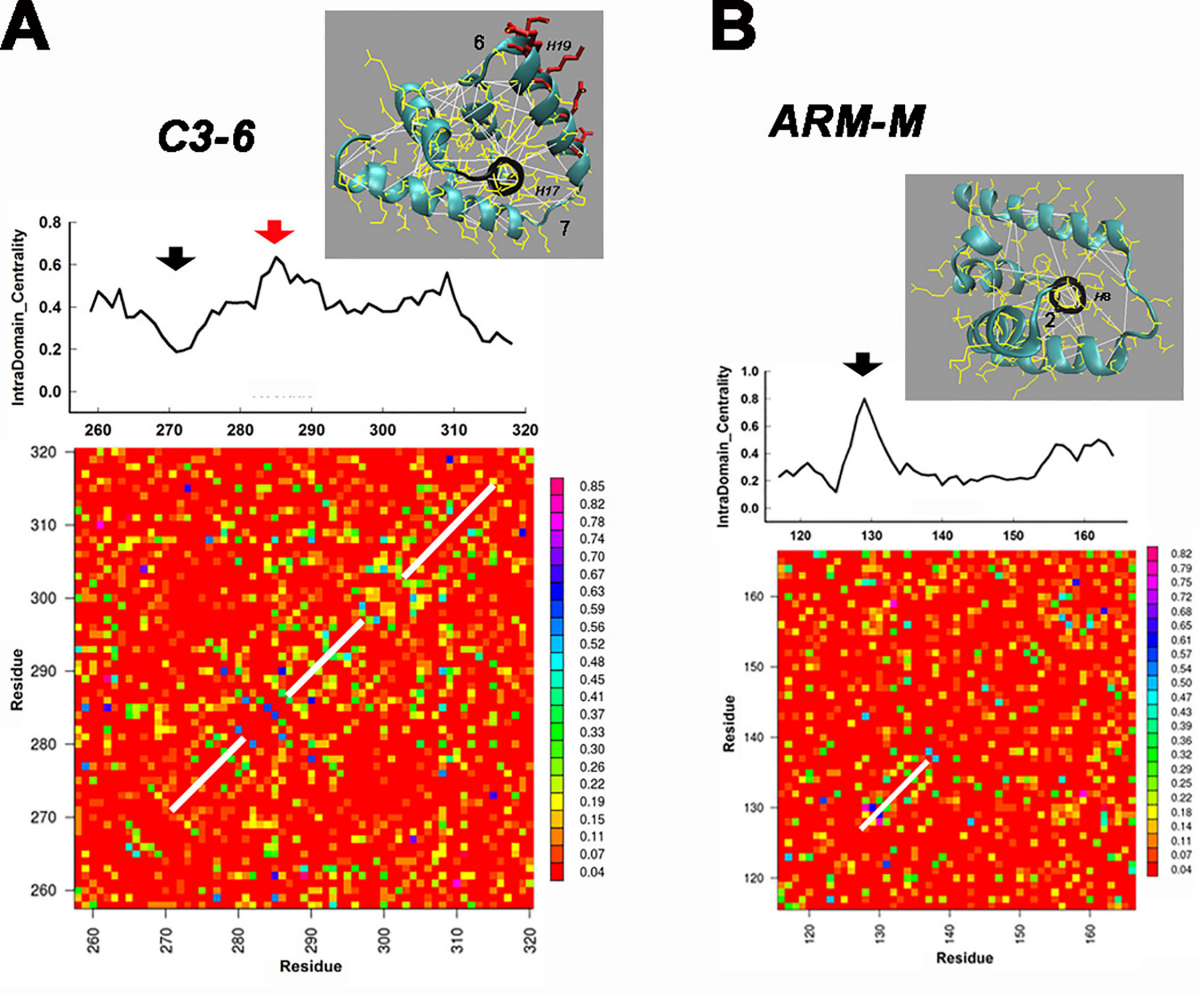
The contact networks of the ARM-M and C3-6 domains. Contact (< 12 angstrom) centrality profiles are positioned on top of their respective coevolution matrices. Matrix segments with white lines along the positive diagonal show the α-helical repeat correlations that generate positive correlations spaced four residues apart, parallel to the white lines. Vertical bars show the colour-coded scale for correlation values as in Fig 3. Numbers in images indicate primary network nodes in the FliG centrality profile (Fig 6A). (**A)** The C3-6 coevolution matrix. The torque helix H19 is the primary node (red arrow) in the centrality profile. The short, hub helix (H17 –black arrow) is adjacent to the linker between the torque-helix and the terminal helix (H20) **Image:** The high-scoring correlations (white lines) mapped onto the 3HJL.pdb C3-6 (cyan backbone, yellow side-chains). H19 (conserved charged residues = red side-chains) and H17 (black backbone) are marked. (**B)** The ARM-M coevolution matrix. Correlations between a short helix (H8 -black) and surrounding helices form a pin-wheel pattern. The H8 helix adjacent to the EHPQ motif forms the primary node (black arrow) in the ARM-M contact centrality. **Image:** The high-scoring correlations mapped onto 3HJL.pdb ARM-M. The correlations and backbones are coloured as in A.

The contact correlations provide insight into internal (“intra-domain”) architecture of the domains. The coevolution matrices for the C3-6 and ARM-M sub-domains are shown together with their centrality profiles (**Fig 7)**. The images (**Fig 7A and 7B)** show the high-scoring correlations mapped onto the 3HJL.pdb fold. A central helix (H8 in ARM-C, H17 in C3-6) in contact with the surrounding helices forms the core of the fold. H1 is the central helix for N1-4 (S1 Fig). In both N1-4 and ARM-M, these helices constitute the primary nodes of the contact networks. The high-scoring correlations radiate out in a pin-wheel pattern from these hub-helices. In C3-6 the pin-wheel is disrupted and the hub-helix (H17) no longer forms a primary node, even though its internal helix contacts are conserved. Instead, the primary node is now the torque helix (H19). The conserved α-helical architecture of the torque helix and adjacent helices, as well as the ARM-M hub adjacent to the EHPQ motif, is evident as bands four residues apart along the diagonal in the matrices (Fig 7). The loops connecting the torque helix to adjacent helices constitute nodes 6 and 7. The ARM-M hub-helix is adjacent to the dominant EHPQ node 2. The contrast between N1-4 and C3-6 is of interest since both sub-domains have a similar fold [6]. It argues that the torque helix is pivotal to coevolution of the C3-6 fold.

### A three-node FliG_M_ FliG_C_ inter-domain network links the EHPQ motif to the C3-6 fold

**Fig 8:**
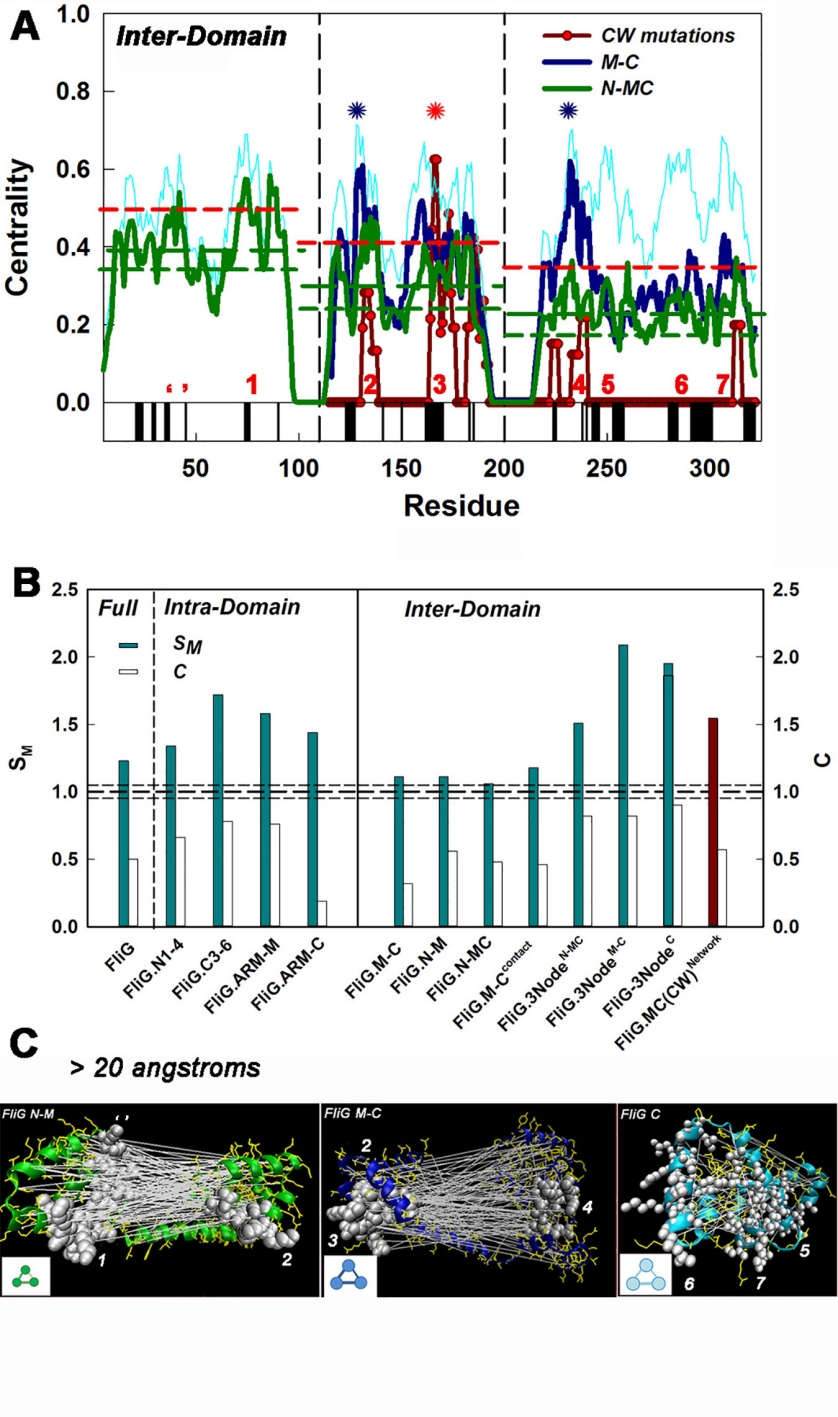
FliG domain interactions. **(A**) Centrality profiles of sub-matrices comprising inter-domain interactions between FliG_N_ and FliG_MC_ (green line) and between FliG_M_ and FliG_C_ (blue line). Residue numbers are as in Fig 6Aii. Cyan line is the complete FliG centrality profile, while the numbers (red) mark its primary nodes Red line with symbols shows the centrality profile of the network constructed from the CW mutations reported in *Salmonella*. Vertical dashed lines demarcate domains; horizontal lines show deviations expected from the randomized library distribution (+2σ (red), -σ (green)) for the FliG_N_FliG_MC_ interaction network. Black bars represent conserved surface residues as in Fig 4A. The dominant nodes for the FliG_M_FliG_C_ interaction (blue asterisks); and the CW network (red asterisk) are marked. **(B)** Bar plots of the S_M_ and C of the complete, intra-domain and inter-domain FliG networks. The values have been normalized relative to the randomized library (mean (thick dashed line) ± σ (thin dashed lines)) as in Fig 5B. (**C)** The high-scoring long-range (>20 angstrom) correlations (white lines) mapped onto the 3HJL.pdb domains. The C^α^ backbone segments are coloured according to the centrality profiles in A, The numbers denote the three nodes, the white spheres the node residues and yellow side-chains other correlated residues. **Insets** (bottom left panels) show relative S_M_ (circle diameter) and C (line thickness) of the 3-node networks.

FliG inter-domain networks were characterized by isolation and analysis of off-diagonal blocks within the complete matrix to define domain interactions. Their centrality profiles were compared against the complete FliG profile (**Fig 8A**). The nodes for the FliG_M_FliG_C_ interaction network superimposed with the complete network primary nodes 2, 3 4, with a weaker contribution from primary node 7. The nodes were localized at or close to the surface. *E*. *coli* cross-link data document the surface proximity of nodes 3 and 7 through formation of FliG oligomers (Table 1). The same three nodes were also the target for CW mutations in *Salmonella*, as discerned from the centrality profile of the CW network. Dummy lists were constructed to evaluate statistical significance. The CW network and the dummy lists were both constructed as for FliM_M_, The mean S_M_ / C of the CW dummy list were 0.79±0.21 / 0.94±0.17 respectively of the real CW FliG_Mc_ network. The mean S_M_ / C of the CCW dummy list were 0.75±0.45 / 0.78±0.30 respectively of the real CCW network. The large standard deviations reflected the greater heterogeneity of the FliG_MC_ coevolution matrix, as compared to FliM_M_. Few CCW mutations have been documented in *Salmonella* FliG_MC_ and they were not considered further.

Primary node 1 within FliG_N_ and an adjacent surface segment formed nodes for interactions with FliG_MC_ (Fig 8A). The interactions are not expected from the structure of the *A*. *aeolicus* full-length FliG in which FliG_N_ is separated by an intervening long helix from the rest of the protein. The long-helix may not be a common feature since it is formed, in part, by a deletion-tolerant sequence segment. Cross-link data indicate that FliG_N_ is in spatial proximity to FliG_M_ in *E*. *coli* [28], consistent with this idea. The mean FliG_C_ W was notably less than for FliG_M_ in the FliG_N_ and FliG_MC_ interaction network centrality profile, No nodes were identified within the FliG_C_ section of this profile.

### The major interactions of the FliG signal transmission pathway

Computation of the S_M_ and C of the FliG short and long-range interaction networks followed the examination of the node weights above. The parameters are listed in **Table 3**and the results are summarized as a bar chart (**Fig 8B**). Among the short-range, intra-domain networks, that for C3-6 has both the greatest S_M_ and C; notably greater than the corresponding metrics for N1-4. The normalized S_M_ value for C3-6 is comparable to FliM_M_, though C is lower. The ARM-C connectivity, C (19% of the randomized library value), is markedly worse than for the other modules.

**Table 3:** FliG Coevolution Matrix.

	ΣMatrix_corr>0_	N_corr_	N_corr>0_	N_Matrix_	N_corr_/N_Matrix_	ΣMatrix_corr>0_/N_corr>0_	C	S_M_
**FliG**_**Randomized**_	11423.3	76887	34532	103041	0.74	0.33	1	1
**FliG**	9314.1	38918	22882	103041	0.38	0.41	0.5	1.23
**FliG_N1-4**	623.2	2134	1402	4356	0.49	0.44	0.66	1.34
**FliG_C3-6**	832.5	2300	1466	3969	0.58	0.56	0.78	1.72
**FliG_ARM-M**	485.7	1470	928	2601	0.57	0.52	0.76	1.58
**FliG_ARM-C**	79.1	236	166	1681	0.14	0.48	0.19	1.44
**FliG**_**M**_ **FliG**_**C**_^**inter**^	810.4	4002	2201	17010	0.24	0.37	0.32	1.11
**FliG**_**N**_ **FliG**_**M**_^**inter**^	745.1	3563	2033	8505	0.42	0.37	0.56	1.11
**FliG**_**N**_ **FliG**_**MC**_^**inter**^	1564.9	8019	4484	22155	0.36	0.35	0.48	1.06
**FliG**_**M**_ **FliG**_**C**_^**Contact**^	45.9	200	117	576	0.35	0.39	0.46	1.18
**FliG**_**N**_ **-FliG**_**M**_ **(3Node)** ^**)inter**^	77.2	264	155	432	0.61	0.5	0.82	1.51
**FliG**_**M**_ **-FliG**_**C**_ **(3Node)** ^**inter**^	102.3	264	148	432	0.61	0.69	0.82	2.09
**FliG**_**C**_ **(3Node)** ^**inter**^	128.8	290	200	432	0.67	0.64	0.9	1.95
**FliG**_**MC**_ **(3Node)** ^**CW-Network**^	33.7	110	66	256	0.43	0.51	0.57	1.54

Parameters used for computation of the strength and connectivity of the FliG networks. Parameter definitions are as in Table 2.

The FliG domain interaction networks have S_M_ values that are lower than for the intra-domain networks, being only marginally greater than the mean S_M_ for the randomized networks. The C values are two-fold lower than those for the intra-domain networks. These S_M_ differences are consistent with the stronger correlations seen between contact pairs (Fig 6C) that mainly represent intra-domain couplings. The FliG_M_FliG_C_ stacking contact observed in some structures (3AJC.pdb, 4FHR.pdb) has somewhat higher S_M_ than the overall FliG_M_FliG_C_ interaction network, analogous to the FliM_M_FliG_M_ contact. However, its correlations are uniformly distributed over the contact helices (S1 Fig), in contrast to the FliM_M_FliG_M_ contact (Fig 5A).

We constructed networks from the top three primary nodes (“3-node networks”) for the long-range networks to evaluate whether these formed the major determinants for the inter-domain interactions. This is the case. The FliG_M_ and FliG_C_ interaction is the strongest. The 3-node network of the FliG_N_ interaction with FliG_MC_ has 1.5 fold greater S_M_ than the complete interaction network, while the 3-node FliG_M_ and FliG_C_ interaction network S_M_ is 2-fold greater (Table 3, Fig 8B). FliG C3-6, with correlations between nodes 6 and 7 (adjacent to the torque helix) and node 5 (H15 just after ARM-C), has the long-range (> 20 angstroms) network with the best connectivity, C, to complement its strong contact network; while its S_M_ is comparable to the 3-node FliG_M_ and FliG_C_ interaction network. The 3-node (2, 3 and 4) CW network too has improved strength and connectivity (Fig 8B, Table 3). The 3-node networks have comparable S_M_ but lower C values relative to the FliM_M_ domain (Table 2). The C value for C3-6 (0.9 (Table 3)) is closest to that for FliM_M_ (0.95 (Table 2)). The high-scoring correlations for the 3-node networks are mapped onto the structures in **Fig 8C**. The topology makes a contact-based rationale for the inter-node correlations improbable, though contacts may occur as a consequence of mobility [15] as considered in Discussion.

In summary, the covariance analysis identifies a pathway for signal transmission from the EHPQ motif to the torque helix. The pathway is built from a patchwork of inter-connected nodes (2, 3 and 4). Node 4 contains the MFXF motif that dominates the sparsely connected ARM-C network module. The sparse ARM-C connectivity suggests that conformational heterogeneity, seen in the superimposed X-ray structures (Fig 6B) smears out residue correlations. Based on both short and long-range correlations, C3-6 forms a conserved fold. A conserved C3-6 fold is in line with the hypothesis, based on the *H*. *pylori* FliG_MC_ structures [15], that FliG_C_ C1-6 responds as a unit to conformational changes within FliM_M_ triggered by CheY. These changes must be relayed, in part, via the EHPQ ARM-M hub (node 2).

### The coevolution of FliG_M_ with FliG_C_ is detected by phylogenetic tree similarity

**Fig 9:**
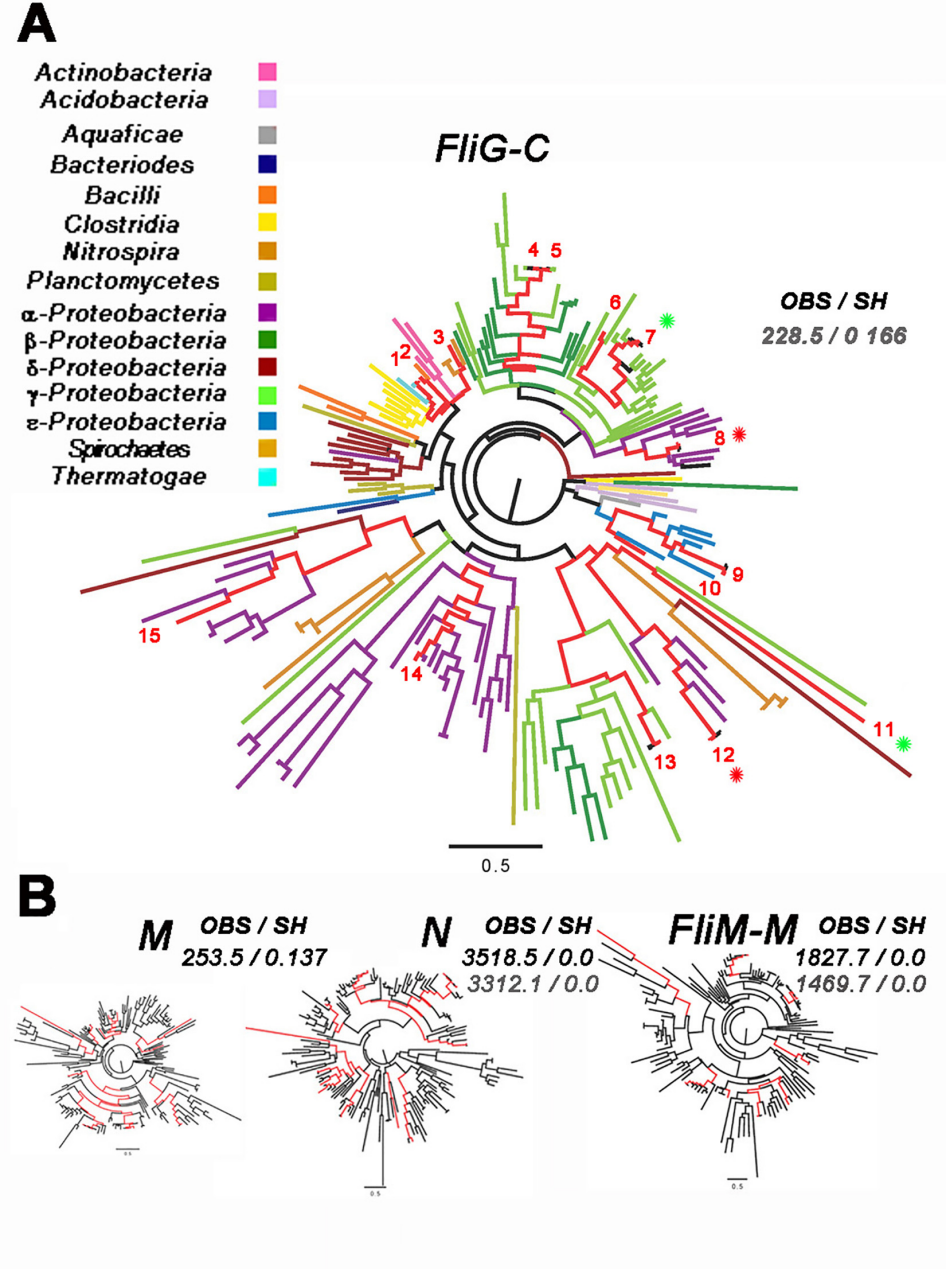
Evidence for phylogenetic similarity between FliG_M_ and FliG_C_. (**A**) Phylogenetic tree of the FliG_C_ domain. 160 seed sequences (duplicate FliG sequences from 23 species). Different phyla are colour coded. γ-proteobacteria are mixed with β-proteobacteria. Numbered representative species (red lines), whose flagellar biochemistry, physiology or structure have been studied are spread round the tree (1 = *Thermotoga maritima*, 2 = *Bacillus subtilis*, 3 = *Borrelia burgdorferi*, 4 = *Escherichia coli*, 5 = *Salmonella typhimurium*, 6 = *Vibrio cholerae*, 7 = *Vibrio alginolyticus1*, 8 = *Rhodobacter sphaeroides1*, 9 = *Helicobacter pylori*, 10 = *Aquifex aeolicus*, 11 = *Vibrio alginolyticus2*, 12 = *Rhodobacter sphaeroides2*, 13 = *Vibrio parahaemolyticus*, 14 = *Caulobacter crescentus*, 15 = *Rhizobium meliloti*). Asterisks (*R*. *sphaeroides* (red), *V*. *alginolyticus* (green)) mark duplicates. (**B)** FliG_M_, FliG_N_ and FliM_M_ phylogenetic trees. Red lines denote the same species as in A. Total branch length: FliG_C_ = 38.6, FliG_N_ = 45.4, FliG_M_ = 40.8, FliM_M_ = 40.0. The similarity measures are OBS, the log-likelihood difference and SH, the probability (0 to 1) that the tree is more similar to the reference tree than the bootstrap replicates. The reference trees were FliG_C_ (black numbers) and FliG_M_ (gray numbers).

We constructed the phylogenetic tree of the FliG_C_ domain to, first, learn more about its evolution (**Fig 9A**). The FliG_C_ phylogenetic tree was colour coded to assess clustering. While both monophyletic and paraphyletic branches were observed, the former were predominant. The firmicutes were the most, and the δ-proteobacteria the least, monophyletic. The α-proteobacteria were the most paraphyletic; consistent with their diversity. The monophyletic branching was consistent with the neutral model of molecular evolution that posits that neutral mutations due to genetic drift are retained with selection based on phenotype while deleterious ones are rapidly eliminated ([39] and references therein). The clustering was disrupted by presence of multiple FliG orthologues in the domain Pfam seed set used for construction of the tree, including two with duplicate flagellar systems in the set of commonly studied species. In some cases, possibly due to horizontal gene transfer, one orthologue localized to a branch for another phylum (eg. *V*.*alginolyticus*). In other cases a phylum (eg. α-proteobacteria) was partitioned between disconnected branches with representatives (eg. *R*. *Sphaeroides*) divided accordingly.

Second, phylogenetic tree similarity offered an independent alternative, with metrics limited by different factors, to check that S_M_, was greatest for the interaction of FliG_M_ with FliG_C_. For the similarity comparison, the FliG_C_ seed sequence MSA was used to extract matching FliG_N_, FliG_M_ and FliM_M_ sequences from the corresponding MSAs in the Pfam database (Methods). For species with multiple FliG orthologues, the single FliM sequence was paired with each FliG sequence. The FliG_C_ tree was the most compact in terms of branch length, consistent with C3-6 residue coevolution (**Fig 9B**). Domain phylogenetic tree topologies were compared in duplicate for each of two reference trees (FliG_M_ and FliG_C_) to check for self-consistency. Coevolution between FliG_C_ and FliG_M_ was detected regardless of choice of reference tree, while coevolution of these domains with either FliM_M_ or FliG_N_ was not. The sensitivity of similarity measures scales with sequence length and is possibly compromised by the short domain sequences. In any case, similarity detection between the FliG_C_ and FliG_M_ trees supported the evidence from the covariance analysis that the interaction between FliG_C_ and FliG_M_ was the strongest.

## Discussion

**Fig 10:**
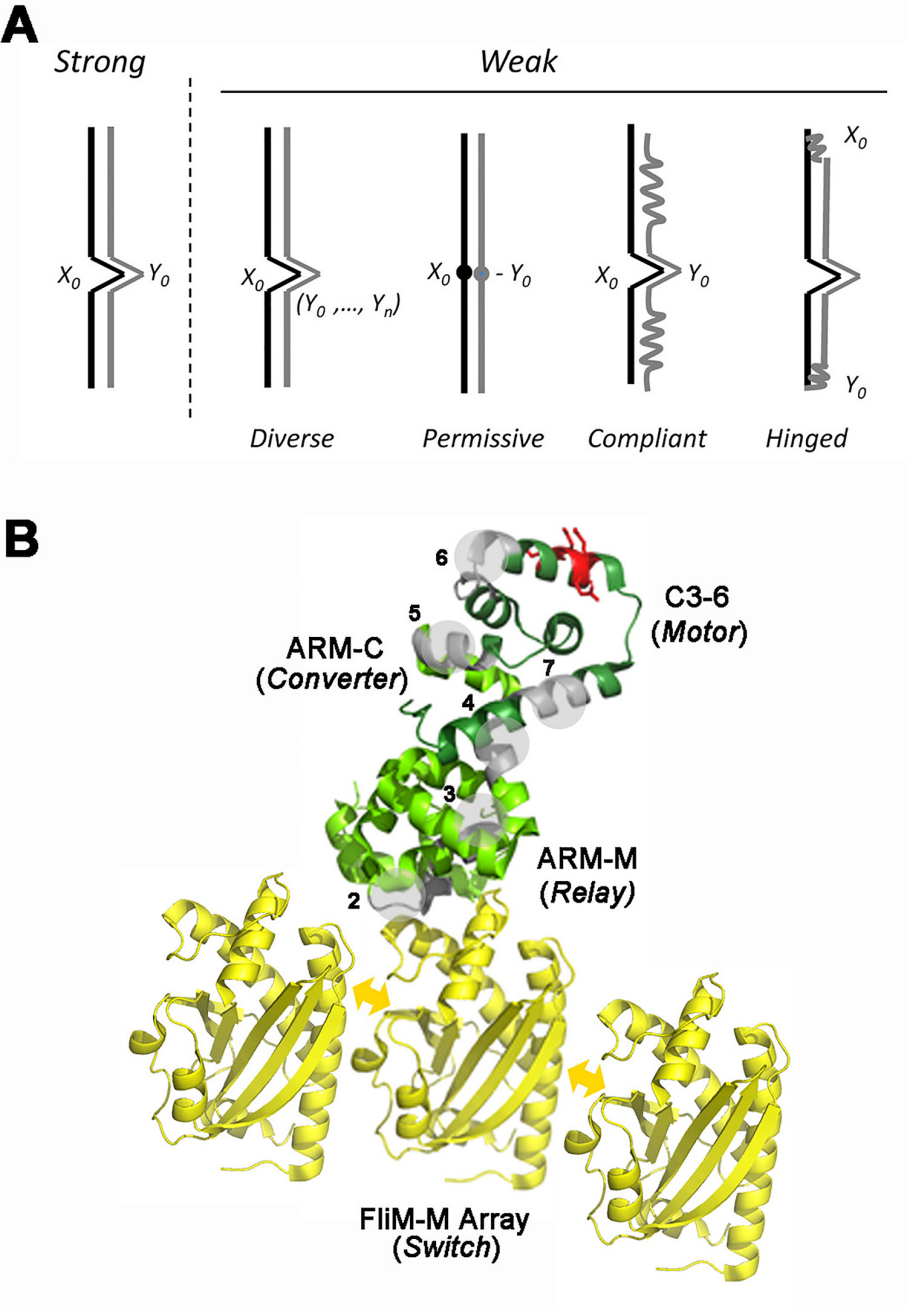
Phylogenetic network architecture of the flagellar motor switch. **A.** Correlation strength depends on contact type. Strong correlation is expected for contacts with hard-wired steric or electrostatic constraints. Change of one residue (X_0_) causes change in a unique partner (Y_0_) to preserve fold. Contacts that produce weak correlations fall into four groups. Diverse: X_0_ has multiple partners due to conformational heterogeneity, or variable subunit symmetry in the case of surface residues. Permissive: X_0_ tolerates multiple partners due to absence of strong constraints. Only certain residues that disrupt the contact interface are forbidden. Compliant: Y_0_ is part of a structural element that is mobile or subject to local denaturation (“melting”). Hinged: X_0_ and Y_0_ are hinge elements coupled via a chain of residues. Alteration in one hinge triggers compensatory change in the other to preserve orientation. **B.** Signal transmission in the flagellar switch complex. The FliM_M_ (gold backbone) fold and inter-subunit contacts are both important for its function. Arrows (gold) denote conformational spread in the FliM_M_ array. The FliG C3-6 motor sub-domain (dark-green) is organized around the torque helix (charged residues (red)). The rest of FliG (light green) is composed of ARM-M and the ARM-C sub-domain. The primary nodes (numbered grey segments overlaid by circular patches) form a relay of allosteric sectors. ARM-C could be the converter element that generates different motor responses from a common switch transition.

We have determined residue coevolution for FliM_M_ alone, FliG alone and FliM_M_FliG_MC_ in complex. We separated intra-domain from inter-domain correlations, identified inter-subunit associations, and assessed network disruption by chemotactic lesions. We developed metrics based on network analysis to measure the correlations. We cannot presently relate the metrics to biochemical parameters such as binding affinity because the coevolution signal may be modulated by a number of factors as illustrated in **Fig 10A**. PSICOV and related algorithms have been optimized to detect hard-wired, native contacts based on static electrostatic or steric constraints, but a large macromolecular assembly such as the switch complex is likely to form a conformational ensemble with diverse dynamics. However, guided by the structural data, we are able to provide a description of the flagellar switch architecture that reveals both common elements as well as possible sources of mechanistic and species diversity.

### The FliM_M_ array forms a concerted switch element

The extended network connectivity of FliM_M_ indicates the importance of the FliM_M_ fold as well as self-association. We take a high mean correlation strength, S_M_, and connectivity, C, of short-range contact correlations as indicators, most simply, of a compact structural fold that is conserved over species. Our data are consistent with molecular dynamics simulations that reveal the high mechanical stability of α/β/α sandwiches [40]. They are also in line with models that propose a central role for FliM_M_ in triggering switching of rotation sense [20,28]. Monte Carlo simulations of conformational spread in the multi-subunit c ring have shown strong coupling between subunits is required to generate the observed two-state switching behaviour [8]. The conserved FliM_M_ inter-subunit contacts suggested by the long-range correlations are consistent with this requirement and, furthermore, identify FliM_M_ as the key determinant for the proposed conformational spread.

The contacts are known targets for *che* mutations [5]. They seem to be stabilized for the conformation representative of the *Salmonella* CCW rotation state, as they are disrupted to a greater extent by CW mutations. Three of the four nodes in the CW mutation coevolution network map to segments previously implicated in FliM self-association. The role of the fourth node is presently unknown. The interfacial surface covered by the coevolved contacts is large. So switching would be attenuated, but not determined by the variations in subunit stoichiometry or localization of the CheY binding sites.

### A dedicated motor module

The FliG_C_ domain (C3-6) based on its coevolved network as measured by all three metrics (W, S_M_ and C), also has a compact fold. The torque helix H19 is central to the C3-6 coevolution network. The H10 contact correlations modify the pin-wheel architecture found for the other FliG ARM domains. This knowledge supplements the conservation of its charged residues responsible for designation of H19 as a torque helix. For torque helix movements to be entrained to C1-6 global motions [15], it needs to be immobilized by contacts with adjacent helices. Our analysis implies this is the case. Accordingly, we propose that the C3-6 sub-domain has been dedicated for motor function.

Primary nodes 6 and 7 flank the torque helix (Fig 7A) and interact strongly among themselves (Fig 8B and 8C). Node 6 is a binding target for the c-di-GMP binding protein YcgR [41] in presence of c-di-GMP, a molecule that regulates several cellular behaviours. Cross-link data indicate that node 6 residues from neighbouring subunits form adjacent surface patches [4] that may function as allosteric sectors (see below). It will be of interest to determine whether node 6 serves as hinge to control C3-6 movements in response to chemotactic stimulation.

### Relay of allosteric sectors

The primary nodes of the coevolved FliG_M_ and FliG_C_ interaction network are the third feature of the common switch architecture. These nodes could constitute an allosteric relay. Studies on dihydrofolate reductase as a model system have shown that inter-connected surface sites, termed “sectors”, are preferred locations for allosteric control. These sectors were hot-spots for deleterious mutations [42]. The primary nodes that wire the EHPQ motif to the C3-6 motor domain have the properties observed for the dihydrofolate reductase sectors; namely distributed spatial organization that, in this case, wires the torque helix to multiple distant surface patches. YcgR may then act as allosteric effector. Furthermore, adjacent subunits could play a similar role in the multi-subunit assembly. Cross-links between residues in nodes 2 and 3 and within node 6, result in the formation of *E*. *coli* FliG oligomers. The *E*. *coli* cross-links could document mobility, analogous to the cross-links between nodes 4 and 7 in *H*. *pylori* (Table 1), consistent with transient association of adjacent subunits for allosteric regulation through freezing out of motions [43]. The dominant EHPQ motif node 2, adjacent to the ARM-M hub helix H8, forms one nexus of a two point FliM_M_FliG_M_ contact. Node 3 includes the GGXG motif and a large conserved surface patch. Node 4 in ARM-C contains the MFXF motif [15]. Nodes 2 and 3 also interact with node 1 in FliG_N_. The relevance of the FliG_N_FliG_M_ interaction for the switching mechanism, if any, is not known. The conservation of the motifs as well as the fact that they were targeted by CW chemotactic mutations was prior knowledge. Their coevolution is the new knowledge revealed by the present study.

Phylogenetic tree similarity measures provide independent support for FliG_M_ coevolution with FliG_C._ The detection of allosteric contacts by covariance analysis is a debated topic [44], since multiple allosteric pathways exist within protein domains [45]. We favour the possibility that signal transmission between FliM_M_ and C3-6 is mediated by allosteric inter-node couplings, but further work is needed, in particular protein dynamics [46], to elucidate these couplings.

### Sources of mechanistic and species diversity

The ARM-C sub-domain is an element of particular interest since, although its MFXF motif (node 4) is integral to FliG network architecture, the sub-domain has sparse connectivity. Multiple factors can contribute (Fig 10A), but the structures suggest an explanation. ARM-C is characterized by conformational heterogeneity within and between species (Fig 6B). Segments of this domain are deleted in many species, while the helix linker connecting ARM-C to ARM-M has segments that could not be resolved in a number of X-ray structures. This linker is truncated or absent altogether from many sequences in the MSA, as is the linker between FliG_N_ and ARM-M, and could also contribute to species diversity. ARM-C must report changes in FliM_M_ conformational state triggered by CheY to C3-6, either via FliG_M_ [17] or directly [20]. The coevolution signal for the ARM-M ARM-C stacking contact [28] seen in some *T*. *maritima* structures was weak relative to ARM-C ARM-M primary node interactions. There was also no signal for the *E*. *coli* ARM-C interaction with FliM_M_ documented by numerous lines of evidence [17,23,28]. The coevolution signal for dynamic contacts may be smeared out by the ARM-C conformational heterogeneity due to the flexible loops. The heterogeneity may generate an ensemble of states from two (CW and CCW) FliM_M_ states, as argued [47] to account for the diversity in motile behaviour seen across species.

A second element that may contribute to diversity is the contact between FliM_M_ and FliG_M_. The contact is built from two FliM_M_ residue segments in the loop at the pseudo-symmetry centre of the domain in both the *T*. *maritima* and *H*.*pylori*, structures [14] A two-point contact with flexible spacing provided by the loop accommodates the variable FliM stoichiometry [48], as well as participation of different protein components. Many species with multiple flagellar systems, for instance those identified in Fig 10, have duplicate *fliG* genes whose products must both associate with a single FliM. Furthermore, FliM subunits may contact FliG_C_ as well as FliG_M_ within the C ring, as proposed for the *E*. *coli* flagellar motor [20,49]. Finally, FliY may also contact FliG in addition to FliM in species that have both proteins, *H*. *pylori* for example. Strong contact between FliM and FliG is not required if the FliM_M_ inter-subunit contacts are conserved in the common switch design to ensure conformational spread. FliG subunits can then be mobilized by the cooperative transition along the FliM_M_ array to report FliM_M_ conformational state to the proximal FliG C3-6 motor domain.

Our conclusions are summarized in **Fig 10B**. FliM_M_ and FliG C3-6 form the dedicated switch and motor domains respectively of the switch complex. FliM_M_ self-association is important for its function during chemotaxis, consistent with the proposed role of conformational spread [8]. The FliG ARM-C domain has weak intra-domain connectivity that reflects the conformational heterogeneity captured by the X-ray structures, but its MFXF motif forms a key interaction node. The circuit connecting the switch and motor domains consists of a chain of nodes, of which the EHPQ motif / ARM-M hub helix form the dominant node. The nodes have properties analogous to the sectors described for allosteric networks.

## Methods

The Methods sections correspond to the boxes in Fig 2 that outlines the computational strategy.

### 1. MSA analysis

Sequences and alignments for the FliG_N_ (PF14842), FliG_M_ (PF14841) and FliG_C_ (PF01706) domains, and FliM_M_ (PF02154) were downloaded from Pfam [50]. The full-sequence Pfam alignments (2000–2600 sequences) are based on construction of a HMM from a curated seed alignment with HMMER3 [51] that was subsequently used to search the sequence database. The MSAs were inspected with JALVIEW [52]. The Pfam headers were replaced with the more comprehensive Uniprot (*http*:*//www*.*uniprot*.*org*) headers for concatenation of the unaligned and aligned sequences. MSA quality was assessed by measurement of the Shannon entropy of residue positions (*S*_*i*_).

$$S_{i}=-\sum_{j=1}^{k}{pij.log}_{2}pij$$

where *p*_*ij*_ is the fraction of sequences at residue position *i* occupied by amino acid *j*. The entropy tends to a minimum value as conservation increases. Gaps are treated as another residue. The domain MSAs were downloaded (Pfam) or generated (CONSURF), then concatenated to obtain overall alignments. CONSURF computes residue conservation based on physico-chemical similarity [53] or evolutionary rate reliant on sequence phylogeny [54]. Alignment of the gap regions provided a metric of alignment quality.

### 2. Coevolved mutations

We used the PSICOV (precise structural contact prediction using sparse inverse covariance) algorithm [38] to compute correlations between residue positions. PSICOV employs arithmetic product correction [55] and normalized mutual information (nMI) [56] to minimize the effects of phylogenetic bias. Sparse inverse covariance estimation based on the glasso algorithm [57] minimizes indirect couplings. The mutual information (MI) between two positions (*i*,*j*) in a MSA is the difference between the sum of the Shannon entropy of the individual positions (*S*_*i*_, *S*_*j*_) and their joint entropy, *S*_*ij*_. The correlation measure is the direct information, *Dij*, between two residue positions,

$$Dij=Wij/\sqrt{\left(Wii.Wjj\right)},$$

where *Wij*, *Wii* and *Wjj* are the inverse of the nMI matrices respectively [58]. The distribution of *Dij* values is normalized by subtraction of the mean values in the two columns for the residue positions. The coevolution matrix is formed from the normalized *Dij* values. Shuffling eliminates correlations between residue positions. The comparison of the real correlation value with the distribution of values from a shuffled population provided a statistical estimate of its significance. Significant correlations (“high-scoring” correlations) were taken as those whose *Dij* values exceeded the distribution mean by 3σ, where σ was the standard deviation of the randomized library distribution.

### 3. Network Analysis

The PSICOV coevolution matrices were used to generate a network model, with the residues as nodes and correlations represented by edges. Bio3D [59] was used for computation of the entropy and analysis of model networks. The matrices were analysed with the igraph network library in R (*http*:*//www*.*igraph*.*org*). Their network representations were examined with Cytoscape [60]. The primary nodes of the network were identified as 6 residue segments whose mean weight, W, in the difference centrality exceeded the distribution mean by 2 σ, with σ based on the randomized library distribution.

### 4 & 5. Phylogenetic Tree Topology

Domain coevolution was assessed by phylogenetic tree similarity [61]. We paired the headers of the Pfam FliG_C_ seed sequence MSA (80 sequences) to headers in the full-sequence FliG_N_, FliG_M_ and FliM_M_ MSAs. Approximately maximum-likelihood phylogenetic trees for constructed from the FliG_C_ MSA and each of the paired MSA using Fast Tree [62]. The paired MSAs were then quered to determine the best match to the topology of the FliG_C_ tree. The process was repeated with another tree as reference. The reliability of tree splits was determined from 100 bootstrap replicates. The results were analysed by CONSEL [63]. CONSEL outputs the log-likelihood difference between the reference and query domain MSAs for the reference tree topology (OBS) and the Shimodaira-Hasegawa test probability (SH) that the reference tree topology is generated by the query MSA In contrast to the standard bootstrap probability, SH corrects for bias due to different sequence length. An alternative approach, based on distance matrices between all protein pairs selected from the similarity in residue composition [64] gave similar results, but was not pursued due to its limitations for analysis of paralogs [65].

### 6. Structure based functional analysis

Structures were downloaded from Protein Data Bank. In addition to the FliM_M_FliG_MC_ complex (4FHR.pdb), there were 2 structures of FliM_M_ (2HP7.pdb, 4GC8.pdb), one structure of FliG_C_ (1QC7.pdb), 2 structures of FliM_M_FliG_M_ (3SOH.pdb, 4FQ0.pdb), and 4 structures of FliG_MC_ (1LKV.pdb, 3AJC.pdb, 3USY.pdb, 3USW.pdb). These structures were of the *T*. *maritima* (4FHR.pdb, 3SOH.pdb, 1LKV.pdb, 3AJC.pdb, 2HP7.pdb) or the *H*. *pylori* (4FQ0.pdb, 4GC8.pdb, 3USY.pdb, 3USW.pdb) proteins. The full length *A*. *aeolicus* FliG (3HJL.pdb) structure completed the set. The MSAs were processed to map residue correlations onto structure. For each structure, the associated sequence was added to the Pfam MSA with mafft-add (*http*:*//mafft*.*cbrc*.*jp/alignment/server/add*.*html*). Residue positions absent from, or not resolved in, the structure sequence were eliminated with a custom script. The PSICOV algorithm was modified to output residue type together with residue position. The match for residue type ensured the high-scoring correlations were mapped correctly onto structure. Physical distances between correlated residue positions were computed from the C^α^ atoms coordinates in the maps. The C^α^ backbones of domains and complexes in the structures were superimposed to assess conformational heterogeneity with analysis tools in GROMACS version 4.5.5 [66]. Superposition was based on a common set of equivalent residue positions identified from the MSA. Determine of topology used the POPS web server [67] to detect surfaces based on residue solvent accessibility and estimate surface hydrophobicity / hydrophilicity. Conservation based on evolution rate, computed with CONSURF, in combination with the POPS score filtered for conserved surface patches. Results were visualized in VMD (*http*:*//www*.*ks*.*uiuc*.*edu/Research/vmd* *)* and Pymol (*http*:*//www*.*pymol*.*org/* *)*.

## Supporting Information

S1 Fig Secondary structure nomenclature and variable fold / interface coevolution. (PDF)

## Abbreviations

PSICOV: precise structural contact prediction using sparse inverse covariance
ARM: armadillo domain
MSA: multiple sequence alignment
HMM: hidden Markov model
H: a-helix
EHPQ: glutamate-histidine-proline-glutamine
MFXF: methionine-phenylalanine-any amino acid-phenylalanine
W: node weight
S_M_: edge strength
C: edge connectivity
