# Supplementary material for: Coevolved Mutations Reveal Distinct Architectures for Two Core Proteins in the Bacterial Flagellar Motor
Source: PLoS One. 2015 Nov 12;10(11):e0142407. doi: 10.1371/journal.pone.0142407 (PMC4642947; doi:10.1371/journal.pone.0142407)
Supplement: S1 Fig — (PDF) [file pone.0142407.s001.pdf]

S1 FIGURE

Secondary structure nomenclature and variable fold / interface coevolution. A. Secondary structure elements in 4HR. Nomenclature for $FliM_M$ follows (1). The domains / sub–domains are coloured as in Figure 1. Nomenclature for $FliG_{MC}$ follows (2). **B.** The intra-domain coevolution network of $FliG_N$. The plots show the $FliG_N$ N1-4 centrality profile (thick line) and the $FliG_C$ C3-6 (thin dashed line) centrality profiles. Residue numbers 1-66 = $FliG_N$ 5K-70A; 1-63 = $FliG_C$ 258A-320D. **Inset:** $FliG_N$ N1-4 domain (cyan backbone, yellow side-chains) with hub helix H1 (black backbone). The H1 sequence forms the primary node (arrow) in the centrality profile**.** The white lines are high-scoring correlations as in Figure 7. **C.** The 3AJC.PDB stacking interaction between $FliG_M$ helix H10 (red backbone) and $FliG_C$ helices H13 /H14 (magenta backbone). Coloured lines map residue (yellow side-chains) correlations. Line colour denotes correlation strength (white (strong) – blue (weak)). The uniform distribution of the correlations in the contact interface contrasts with that observed for the $FliM_M$ / $FliG_M$ contact interface.

1. Vartanian, A. S., A. Paz, E. A. Fortgang, J. Abramson, and F. W. Dahlquist. 2012. Structure of flagellar motor proteins in complex allows for insights into motor structure and switching. The Journal of biological chemistry 287:35779-35783.
2. Lee, L. K., M. A. Ginsburg, C. Crovace, M. Donohoe, and D. Stock. 2010. Structure of the torque ring of the flagellar motor and the molecular basis for rotational switching. Nature 466:996-1000.

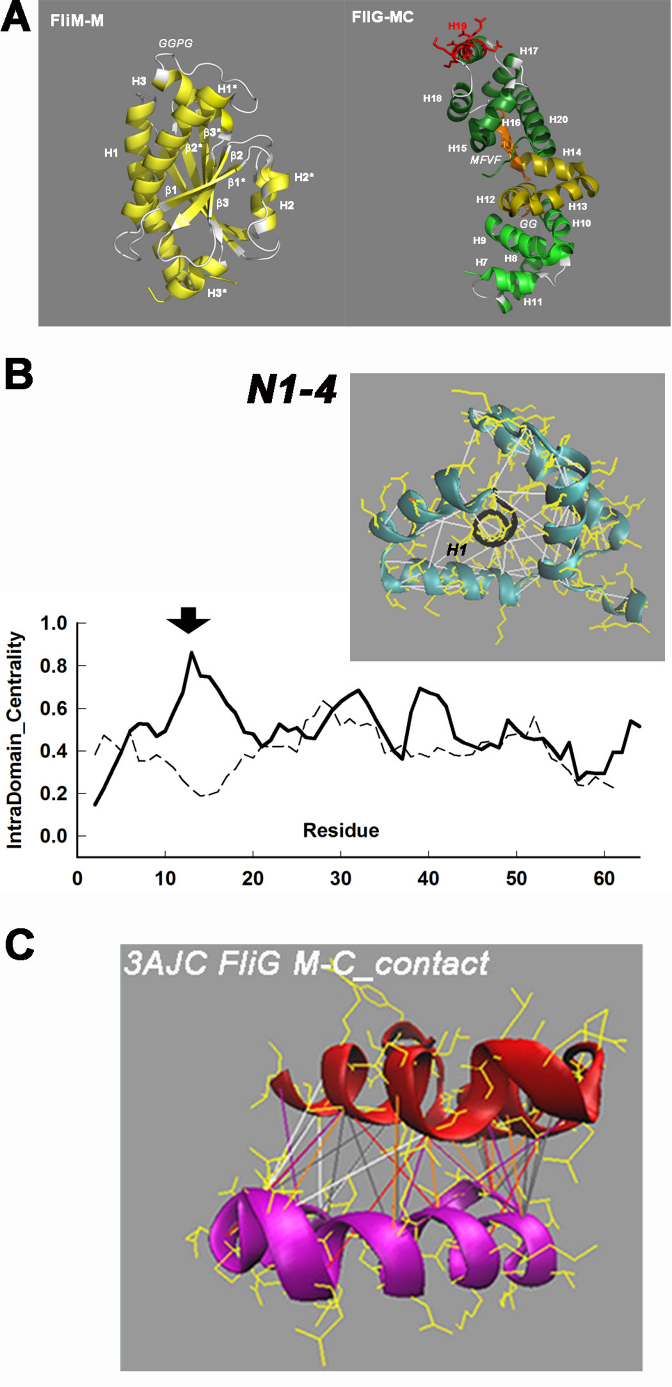
A
FliM-M
GGPG
H3
H1*
β3*
β2*
H1
β2
H2*
β1*
β1
β3
H2
H3*
FliG-MC
H19
H17
H18
H16
H20
H15
MFVF
H14
H12
H13
GG
H10
H9
H8
H7
H11
B
N1-4
H1
IntraDomain_Centrality
Residue
1.0
0.8
0.6
0.4
0.2
0.0
0
10
20
30
40
50
60
C
3AJC FliG M-C_contact

Figure S1
